# Copy Number Variations in Amyotrophic Lateral Sclerosis: Piecing the Mosaic Tiles Together through a Systems Biology Approach

**DOI:** 10.1007/s12035-017-0393-x

**Published:** 2017-01-24

**Authors:** Giovanna Morello, Maria Guarnaccia, Antonio Gianmaria Spampinato, Valentina La Cognata, Velia D’Agata, Sebastiano Cavallaro

**Affiliations:** 1 Institute of Neurological Sciences (ISN), National Research Council (CNR), Via Paolo Gaifami, 18, 95126 Catania, Italy; 2 Department of Biomedical and Biotechnological Sciences, Section of Human Anatomy and Histology, University of Catania, Catania, Italy

**Keywords:** Amyotrophic lateral sclerosis (ALS), Copy number variations (CNVs), Systems biology, Genomics

## Abstract

Amyotrophic lateral sclerosis (ALS) is a devastating and still untreatable motor neuron disease. Despite the molecular mechanisms underlying ALS pathogenesis that are still far from being understood, several studies have suggested the importance of a genetic contribution in both familial and sporadic forms of the disease. In addition to single-nucleotide polymorphisms (SNPs), which account for only a limited number of ALS cases, a consistent number of common and rare copy number variations (CNVs) have been associated to ALS. Most of the CNV-based association studies use a traditional candidate-gene approach that is inadequate for uncovering the genetic architectures of complex traits like ALS. The emergent paradigm of “systems biology” may offer a new perspective to better interpret the wide spectrum of CNVs in ALS, enabling the characterization of the complex network of gene products underlying ALS pathogenesis. In this review, we will explore the landscape of CNVs in ALS, putting specific emphasis on the functional impact of common CNV regions and genes consistently associated with increased risk of developing disease. In addition, we will discuss the potential contribution of multiple rare CNVs in ALS pathogenesis, focusing our attention on the complex mechanisms by which these proteins might impact, individually or in combination, the genetic susceptibility of ALS. The comprehensive detection and functional characterization of common and rare candidate risk CNVs in ALS susceptibility may bring new pieces into the intricate mosaic of ALS pathogenesis, providing interesting and important implications for a more precise molecular biomarker-assisted diagnosis and more effective and personalized treatments.

**Electronic supplementary material:**

The online version of this article (doi:10.1007/s12035-017-0393-x) contains supplementary material, which is available to authorized users.

## Background

**Fig. 1:**
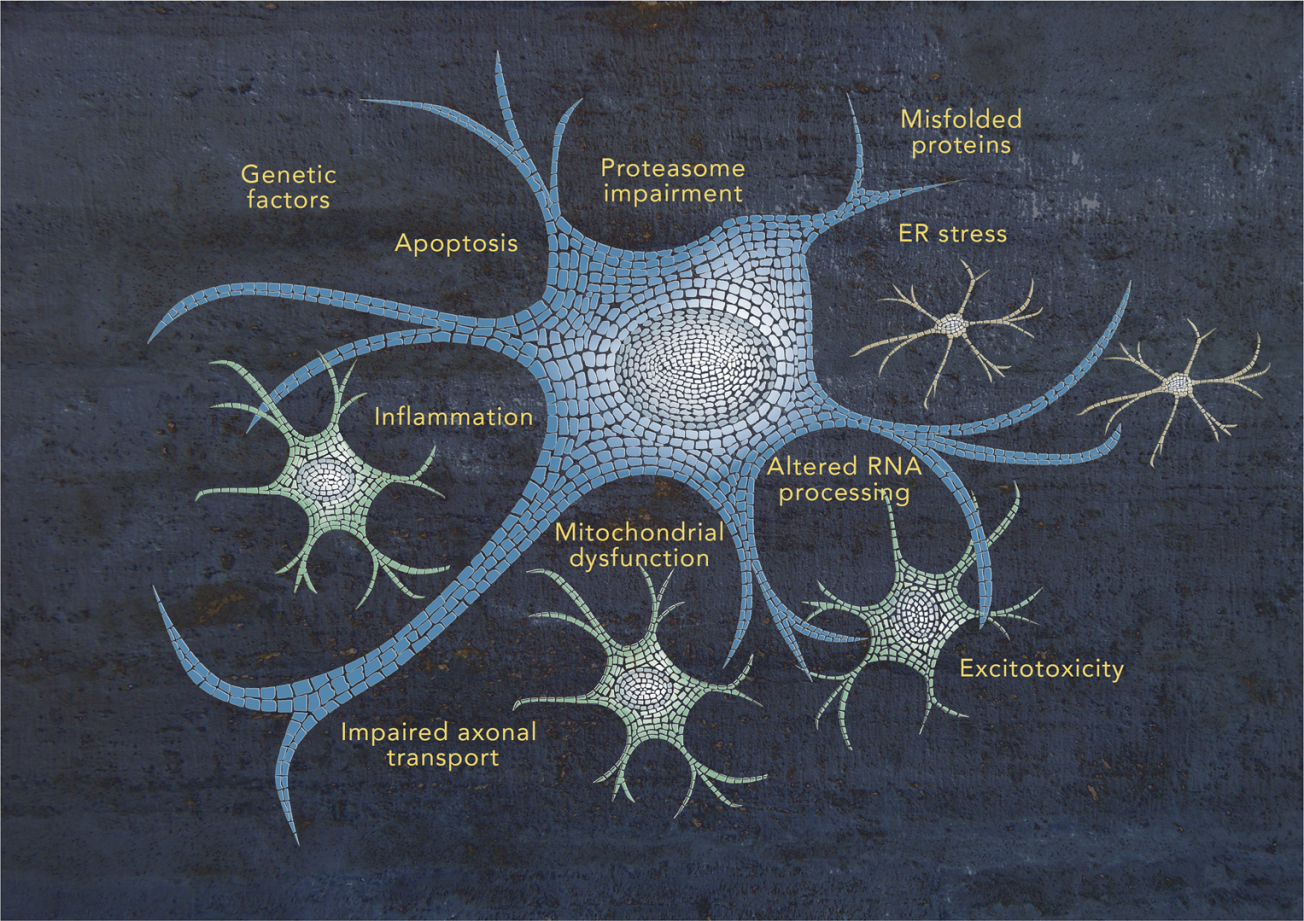
Schematic representation of the complex mosaic of ALS pathogenesis

Amyotrophic lateral sclerosis (ALS) is a devastating untreatable neurodegenerative disease characterized by the selective degeneration of motor neurons in the brain and spinal cord, leading to paralysis and death, usually from respiratory failure, within 3–5 years of onset [1]. The disease exists in two forms: familial ALS (FALS) and sporadic ALS (SALS). FALS is a rare monogenic disease that occurs in 5–10% of cases with an autosomal dominant inheritance and for which several causal genes have been identified, including *SOD1*, *ALS2*, *SETX*, *SPG11*, *FUS*, *VAPB*, *ANG*, *TARDBP*, *FIG4*, *OPTN*, *ATXN2*, *UBQLN2*, *PGRN*, *PFN1*, *DCTN1*, and *C9ORF72* [2]. SALS comprises the majority (90–98%) of ALS cases and is considered to be a complex multifactorial disorder, involving multiple pathogenic processes, such as oxidative stress, protein aggregation, mitochondrial dysfunction, excitotoxicity, and impaired axonal transport (Fig. 1) [3]. Although there are still missing pieces in the intricate mosaic of SALS pathogenesis, several studies have recognized the important contribution of genetic risk factors, usually associated with incomplete penetrance, and gene-environment interactions for disease susceptibility (Fig. 1).

The remarkable advances in genome technologies over the last years have led to huge progress in the understanding of the genetic mechanisms involved in ALS. The search for pathogenic variants has been initially focused primarily on variations at the single-nucleotide polymorphism (SNP) level. In the last years, several candidate-gene or genome-wide association studies (GWAS) have identified multiple SNPs affecting potentially ALS-associated genes, including *VEGFA*, *ANG*, *FGGY*, *DPP6*, *ITPR2*, *KIFAP3*, and *UNC13A* [4–7]. However, the contribution of nucleotide sequence abnormalities in SALS pathogenesis remained unclear, with point mutations in the above genes occurring only rarely.

**Fig. 2:**
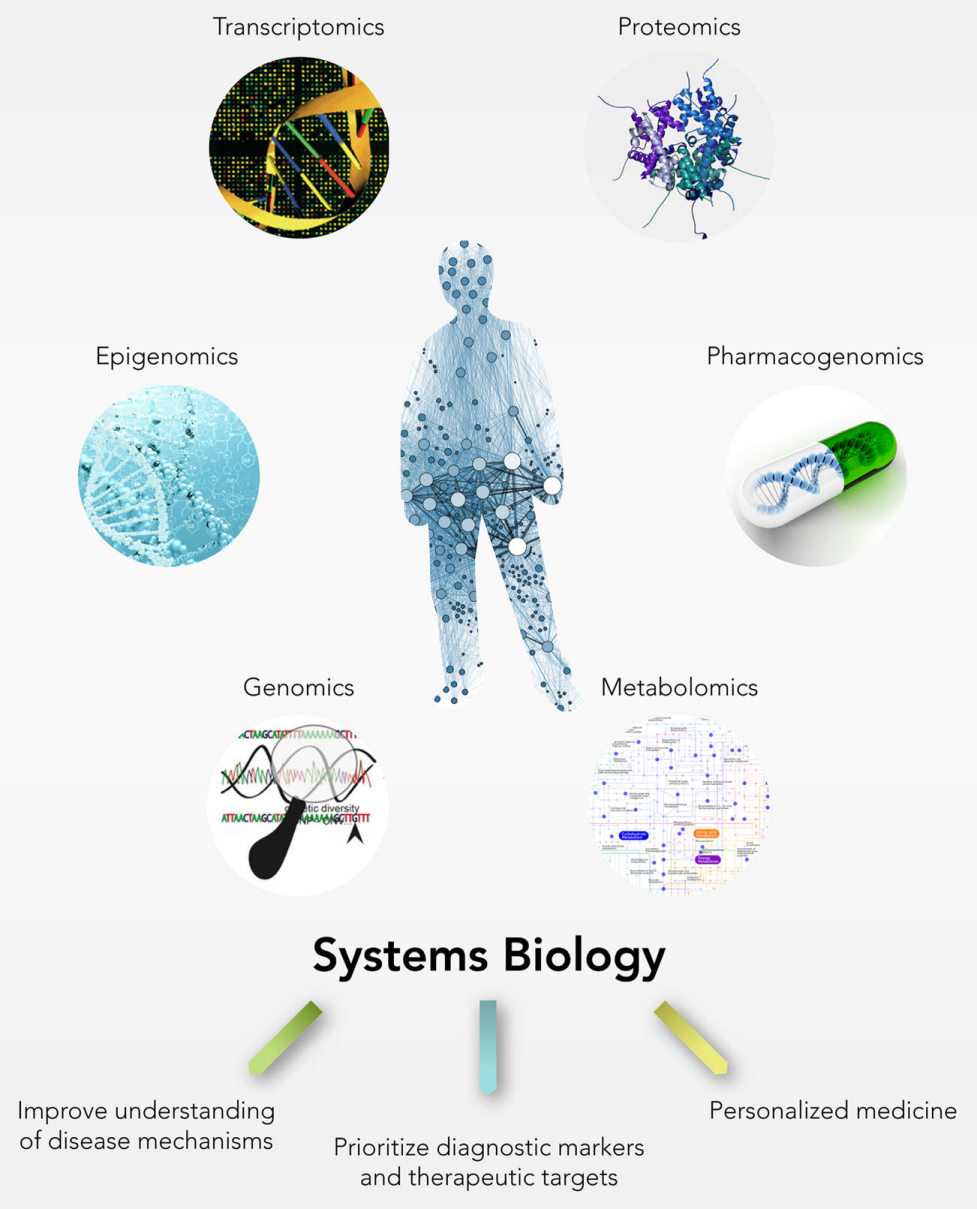
The systems biology approach: from integration of large-scale “omics” data to personalized medicine practice

In addition to SNPs, submicroscopic chromosomal changes, also known as copy number variations (CNVs), represent a substantial source of inter-individual genetic variations exerting important phenotypic effects on the expression and function of genes and representing one of the major risk factors for various complex human disorders including ALS [8, 9]. The majority of existing CNV genotyping studies use a traditional single-gene approach that, albeit has provided valuable information regarding the impact of individual common variants, is inadequate for uncovering genetic architectures of complex traits like ALS, often leading to the loss of less-frequently but potentially functionally relevant CNV-driven gene sets. In this context, the holistic “systems biology” approach provides a new perspective in the study of complex disease traits, which goes beyond the conventional one-gene-at-a-time testing scheme, and embraces the entire equilibrium of a biological system undergoing a much more complicated network of molecular components that in association increase the likelihood of developing the disease (Fig. 2) [10].

In this review, we will explore the currently known landscape of CNVs, putting specific emphasis on CNV-driven genes that have been consistently associated with an increased risk of ALS, considering their potential functional impact in the pathophysiology of the disease. In addition, we will examine the potential contribution of multiple rare CNVs in ALS pathogenesis, focusing our attention on the complex mechanisms by which these proteins might impact, both individually or in combination, to the genetic susceptibility of ALS.

## Main Text

### Copy Number Variations: an Overview

One of the most important scientific discoveries about the human genome study is that, in addition to nucleotide sequence variants such as SNPs, different types of genomic structural variations contribute significantly to genetic heterogeneity [11, 12]. Genomic structural variants include many different types of chromosomal rearrangement encompassing both inversions and balanced translocations as well as genomic imbalances commonly referred to as copy number variations (CNVs).

CNVs are defined as fragments of DNA larger than 1 kb (variants smaller than 1 kb are termed InDels) presenting unbalanced rearrangements in comparison to a reference genome [13–15]. These can be rare (<1%) or common (>5%); de novo or inherited; and include structural gains (duplication or insertional transpositions), losses (e.g., deletions), or complex rearrangements. In particular, deletions can be heterozygous (with only one copy missing), homozygous (with both copies missing), or hemizygous (e.g., X chromosome deletions in males) [16]. In the case of copy number gains, it is possible to distinguish duplications (three copies of a genomic region) from other complex genomic rearrangements, such as homozygous duplications (four copies with two copies on each allele), triplications (four copies with three copies on one allele and one copy on the other allele), and quadruplications (five copies) [17]. Although single-copy deletions and gains of one or two extra copies of DNA are common, the complete genetic loss of both alleles and more extensive amplification of specific DNA regions have been only rarely described.

### Methodological Approaches for CNVs Detection and Analysis

**Fig. 3:**
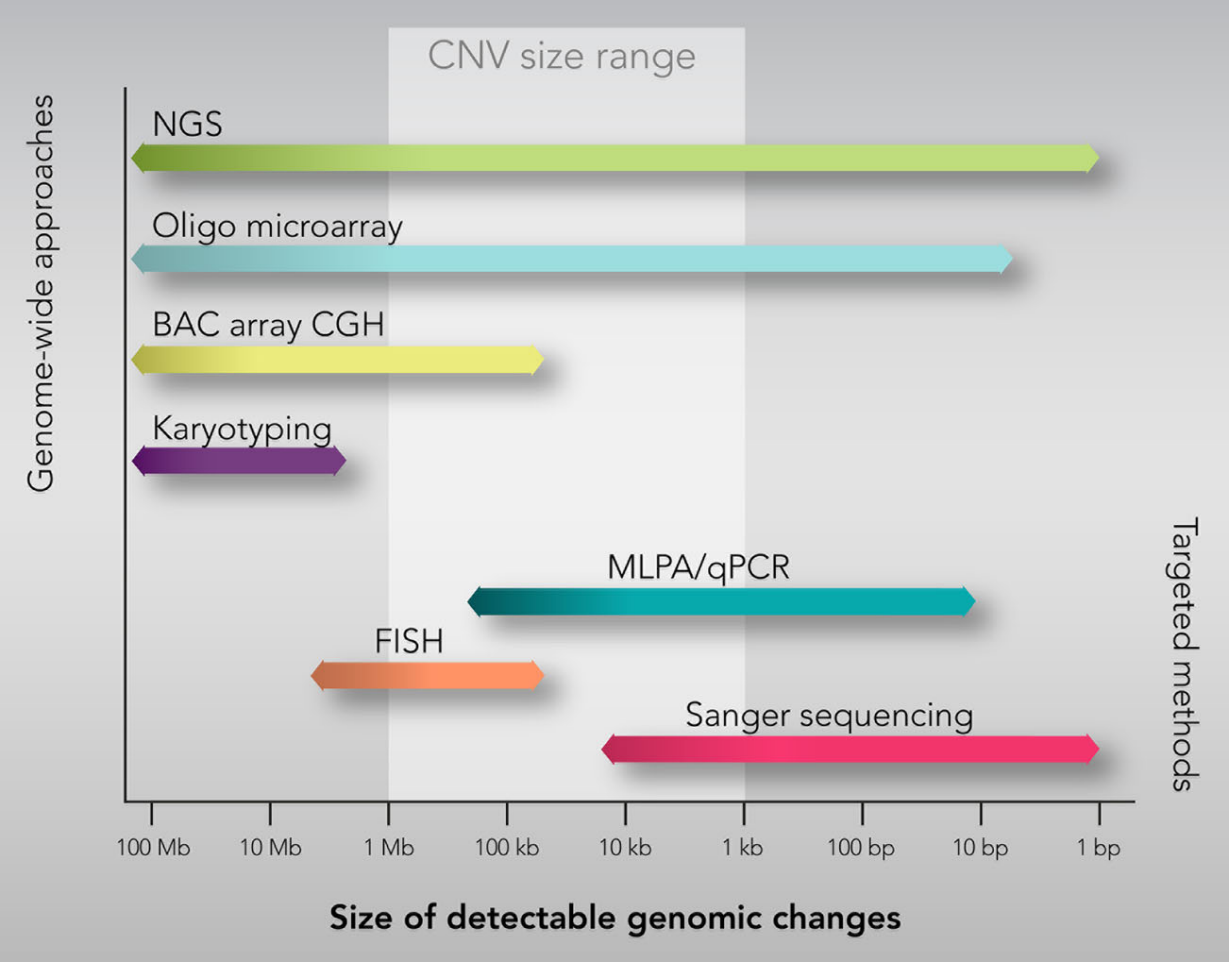
The most widely used methods for genome-wide and targeted CNVs detection and analysis

The conventional karyotyping techniques typically detect chromosomal aberrations greater than 5–10 Mb ruling out other forms of submicroscopic CNVs. The recent advent of genome-wide approaches has driven much of the research on CNVs facilitating their identification and characterization at much higher resolution than hitherto. Several methods for detecting CNVs are currently available and can be categorized into genome-wide and targeted detection approaches (Fig. 3) [18].

Genome-wide approaches permit to scan the entire genome for CNVs detection and include microarray-based (array-comparative genomic hybridization (CGH) and SNP array) and next-generation sequencing (NGS)-based analyses (Fig. 3). Originally, array-CGH included only a few hundreds of thousands of large-insert clones (known as bacterial artificial chromosomes (BACs)) with a relatively low spatial resolution (typically >5–10 Mb) and required a relatively large volume of DNA [19]. Subsequently, high-density oligo-synthesized arrays have been developed, offering a much higher resolution with a more accurate definition of CNV boundaries [20]. In addition to array-CGH, high-density SNP genotyping arrays have also gained interest for CNV detection and analysis, mainly due to their dual role for both SNP- and CNV-based association studies [21]. Although microarray technologies currently represent the most powerful available method to identify CNVs, they cannot detect short CNVs. The advent of NGS technologies has allowed to overcome this limitation, offering the possibility to reveal much smaller CNVs (<10 kb) and define CNV breakpoints at the single nucleotide level [22].

Despite genome-wide approaches providing reliable and efficient methodological insights for large-scale association analyses, other CNV detection methods are preferably used for the validation process as well as phenotype/genotype correlation and clinical translation of a relatively small set of known CNVs. These “targeted methods” include fluorescence in situ hybridization (FISH), quantitative polymerase chain reaction (qPCR), and multiplex ligation-dependent probe amplification (MLPA) (Fig. 3) [14]. FISH has been the first molecular method able to detect submicroscopic genomic CNVs, but it is a time-consuming method that requires prior knowledge of the regions of interest, not allowing for genome-wide analyses [23]. qPCR is an efficient method for screening CNV-targeted genomic regions. However, this technique does not allow the simultaneous amplification and quantification of a large number of targets in a single reaction. MLPA is an alternative targeted PCR-based approach that allows simultaneous analysis of multiple targets (up to 40 targets) with one primer pair, reducing the probability of obtaining spurious qPCR results due to different reaction conditions [24].

### The Origin of CNVs and their Functional Impact in the Human Genome

Significant progress has been made to date in the field of CNV detection and analysis; however, much remains to be investigated about mechanisms that cause CNVs and their functional consequences. CNVs can arise from a multitude of molecular mechanisms (e.g., non-allelic homologous recombination between repeated elements, non-homologous DNA repair mechanisms, replication errors, and transposable element-mediated mechanisms) and can involve one or multiple genes as well as regulatory regions [25, 26]. Based on their size and the genomic region in which they occur, CNVs can affect gene functions in a variety of ways: by disrupting gene coding sequences (for example by deleting or inserting exonic sequences), producing novel fusion genes and splice variants, as well as by affecting regulatory elements and other functional regions, thus perturbing the assembly of the transcription machinery. These variations at the genomic level may result in alterations at the protein level, leading to eliminated/reduced or increased expression levels or interrupted coding sequences that cause the formation of truncated proteins with altered functions. Moreover, disruption of the regions including transcription regulatory elements and enhancers can also result in long-distance effects (even up to 1 Mb) by altering the expression of genes in neighboring chromosomal regions [27].

Changes in copy number are extremely frequent in the human genome (12–15%) representing one of the most prevalent types of genetic variations [11]. Approximately, more than 66,000 CNVs and 34,000 InDels were identified in various populations and cataloged in the Database of Genomic Variants (DGV) (http://dgv.tcag.ca/dgv/app), a public and comprehensive catalog of human CNVs and genomic structural variations identified in healthy controls [8, 28]. While the majority represents benign polymorphic variants, increasing numbers of CNVs are associated with a higher risk of developing various types of inherited Mendelian and complex disease traits, including neurological disorders [29, 30]. Several recent studies, in fact, have supported the role of CNVs in causing or influencing the susceptibility to many neurological and developmental conditions, such as Charcot–Marie–Tooth neuropathy [31], autism [32], schizophrenia [33], epilepsy [34], Crohn’s disease [35], and neurodegenerative disorders, including Alzheimer’s, Parkinson’s and ALS [36, 37].

### Copy Number Variations in ALS

Recent advances in genomic technologies have led to huge progress in understanding the genetic mechanisms underlying ALS, allowing the identification and characterization of novel genetic susceptibility loci, such as CNVs [38]. Various studies have, in fact, highlighted the possible association between common and rare candidate CNV-driven genes or genomic regions and increased risk of developing ALS (Table 1).

**Table 1:** Characteristics of the most significant CNVs and overlapped genes from different genome-wide studies showing association with ALS

Chr	Position start–end (CNV length)	CNV type	CNV detection method	ALS patients	Controls	Overlapped genes^¥^	Reference
1	41,119,815–41,147,030	Gain	SNP array (TaqMan qPCR validation)	8 (no. cases = 406)	1 (no. controls = 404)	None	[115]
1	Not reported (2246 bp)	Deletion	SNP array	3 (no. cases = 12)	0 (no. controls = 24)	Intron of **FMN2**	[138]
1	52,994,160–53,423,907	Gain	BAC array (aCGH validation)	1 (no. cases = 72)	0 (no. controls = 700)	ZYG11B, ECHDC2, SCP2, PODN, **SCL1A7,** CPT2, C1orf23, MAGOH	[165]
3	2,944,819–2,947,844	Gain	SNP array	2 (no. cases = 32)	0 (no. controls = 26 )	Intron of **CNTN4**	[138, 165]
3	89,485,137–89,499,861	Loss	SNP array (TaqMan qPCR validation)	2 (no. cases = 117)	11 (no. controls = 109)	**EPHA3**	[96]
3	33,270,957–33,296,620	Gain	SNP array	3 (no. cases = 575)	18 (no. controls = 621)	FBXL2	[110, 111]
3	Not reported (6527 bp)	Deletion	SNP array	1 (no. cases = 12)	0 (no. controls = 24)	Promoter of **CHL1**	[138]
3	Not reported (53,562 bp)	Loss	SNP array	1 (no. cases = 12)	0 (no. controls = 24)	52% of **DHX30** (+ promoter), 9% of SMARCC1 (+ promoter)	[138]
3	60,357,746–60,604,845	Gain	BAC array (aCGH validation)	1 (no. cases = 72)	0 (no. controls = 700)	FHIT	[165]
4	761,587–1,014,752	Gain	SNP array	16 (no. cases = 575)	4 (no. controls = 621)	**CPLX1**, GAK, TMEM175, DGKQ, IDUA, SLC26A1, FGFRL1	[110]
4	Not reported (144,772 bp)	Loss	SNP array	1 (no. cases = 12)	0 (no. controls = 24)	3% of **RPS3A** (+ promoter), 39% of LRBA (+ promoter)	[138]
4	Not reported (4546 bp)	Deletion	SNP array	1 (no. cases = 12)	0 (no. controls = 24)	Promoter of **UGT8**	[138]
5	28,842,013–28,912,873	Gain	SNP array	7 (no. cases = 575)	0 (no. controls = 621)	None	[110]
5	45,850,032–46,384,240	Gain	SNP array	264 (no. cases = 575)	174 (no. controls = 621)	**HCN1** (flanking)	[110]
5	70,925,030–70,953,012	Gain	MLPA assay	121 (no. cases = 1689)	68 (no. controls = 1780)	**SMN1–SMN2**	[45–47]
		Loss	qPCR	14 (no. cases = 167)	4 (no. controls = 310)		
6	109,034,609–109,074,882	Gain	SNP array	1 (no. cases = 32)	0 (no. controls = 26 )	Intron of **FOXO3**	[99]
6	123,569,244–124,360,902	Gain	BAC array (aCGH validation)	1 (no. cases = 72)	0 (no. controls = 700)	**TRDN,** TCBA1	[165]
7	153,031,806–154,276,435	Loss and gain	SNP array (TaqMan qPCR validation)	10 (9 dup, 1 del) (no. cases = 1875)	13 (12 dup, 1 del) (no. controls = 8731)	**DPP6**	[84]
7	61,663,407–62,155,064	Gain	SNP array	177 (no. cases = 575)	132 (no. controls = 621)	None	[110]
8	47,062,007–47,406,312	Gain	SNP array	30 (no. cases = 575)	8 (no. controls = 621)	**POTEA** (flanking)	[110]
8	47,062,007–47,711,911	Gain	SNP array	3 (no. cases = 575)	18 (no. controls = 621)	None	[110]
8	43,689,385–43,910,848	Gain	SNP array	74 (no. cases = 575)	52 (no. controls = 621)	None	[110]
8	73,609,541–73,629,084	Gain	SNP array	1 (no. cases = 32)	0 (no. controls = 26 )	Region overlaps with 86.50% of **KCNB2**	[99]
8	144,686,338–144,765,210	Loss and gain	SNP array	6 (no. cases = 575)	0 (no. controls = 621)	ZC3H3, **GSDM,** C8orf73, NAPRT1, **EEF1D**, TIGD5, PYCRL	[110]
10	1,050,000–1,090,000	Gain	aCGH (TaqMan qPCR and high-density customized aCGH validation)	46 (no. cases = 83)	10 (no. controls = 100)	**IDI1-IDI2**	[78]
11	50,545,00–50,586,426	Loss	SNP array	21 (no. cases = 117)	2 (no. controls = 109)	None	[96]
11	539,119–652,407	Loss	SNP array	5 (no. cases = 575)	0 (no. controls = 621)	**OR4A5, OR4C12** (flanking) LRRC56, C11orf35, RASSF7, PHRF1, IRF7, MUPCDH, SCT, DRD4, DEAF1	[110]
12	36,528,296–36,801,139	Gain	SNP array	157 (no. cases = 575)	8 (no. controls = 621)	None	[110]
14	103,232,016–103,721,150	Gain	SNP array	5 (no. cases = 575)	0 (no. controls = 621)	KLC1, XRCC3, ZFYVE21, **PPP1R13B,** C14orf2, TDRD9, ASPG, KIF26A	[110]
14	Not reported (4384 bp)	Deletion	SNP array	5 (no. cases = 12)	0 (no. controls = 24)	None	[138]
15	20,387,566–44,672,396	Loss and gain	SNP array (TaqMan qPCR validation)	12 (4 dup, 8 del) (no. cases = 1875)	34 (31 dup, 3 del) (no. controls = 8731)	TUBGCP5, CYFIP1, NIPA2, **NIPA1**	[84]
15	Not reported (5695 bp)	Deletion	SNP array	2 (no. cases = 12)	0 (no. controls = 24)	None	[138]
15	19,818,989–20,084,080	Deletion	SNP array	2 (no. cases = 32)	0 (no. controls = 26 )	LOC650137 (+), **OR4M2** (+), **OR4N4** (+)	[99]
16	76,578,045–77,657,555	Loss	SNP array	0 (no. cases = 406)	6 (no. controls = 404)	CLEC3A, WWOX	[115]
16	87,957,353–87,971,263	Gain	SNP array	2 (no. cases = 32)	0 (no. controls = 26)	Intron of **ANKRD11**	[99]
16	969,913–1,834,962	Gain	SNP array	8 (no. cases = 575)	15 (no. controls = 621)	SOX8, SSTR5, C1QTNF8, **CACNA1H**, TPSG1, TPSB2, TPSAB1, TPSD1, **UBE2I, BAIAP3**, C16orf42, GNPTG, UNKL, C16orf91, CLCN7, C16orf38, TELO2, IFT140, TMEM204, CRAMP1L, HN1L, **MAPK8IP3**, NME3, MRPS34, EME2, **SPSB3**, NUBP2, **IGFALS**, HAGH, FAHD1, C16orf73	[110]
17	Not reported (1159 bp)	Gain	SNP array	1 (no. cases = 12)	0 (no. controls = 24)	**AATK**, ACTG1, AZI1, BAHCC1, **BAIAP2**, C17orf55, C17orf56, C17orf70, C17orf89, **CHMP6**, FSCN2, SLC38A10, TMEM105, 2% of NPLOC4 (+ promoter), 31% of KIAA1303	[138]
19	20,860,930–20,875,787	Loss	SNP array	15 (no. cases = 117)	2 (no. controls = 109)	None	[96]
19	32,615,675-32,935,836	Gain	SNP array	165 (no. cases = 575)	122 (no. controls = 621)	RDH13	[110]
19	Not reported	Loss	SNP array	2 (no. cases = 12)	0 (no. controls = 24)	Intron of **ZFP14**	[138]
22	23,696,411–24,240,667	Loss and gain	SNP array	10 (8 gain 2 loss) (no. cases = 406)	28 (no. controls = 404)	CRYBB3, CRYBB2, LOC91353, LRP5L, CRYBB2P1	[115]
22	21,011,312–21,394,287	Loss	SNP array	0 (no. cases = 575)	11 (no. controls = 621)	ZNF280B, ZNF280A, PRAME, BCR, **GGTLC2**	[110, 138]
22	29,489,697–29,489,738	Deletion	SSCP	5 (no. cases = 530)	2 (no. controls = 379)	**NEFH**	[68]
X	139,400,576–telomere	Deletion	BAC array (aCGH validation)	1 (no. cases = 72)	0 (no. controls = 700)	>100 (No ALS candidate genes)	[165]
X	139,526,743–139,942,807	Gain	BAC array (aCGH validation)	1 (no. cases = 72)	0 (no. controls = 700)	**CDR1**	[165]

The table shows the most significant CNV loci and relative genes that partially or completely fall within them. Chromosomal positions are referred to the human reference genome assembly corresponding to each individual study: in particular [115], [40], and [81] refer to the NCBI reference sequence build 36, [98] refers to the build 38 and [41] refers to UCSC Genome Browser, May 2004 Freeze. In the case of [71], the authors described deletion coordinates by using the numbering of the published sequence by *Lees* et al. *EMBO J. 1988*. Genes that may be reasonable ALS candidates are in bold

*Chr* Chromosome, *SSCP* single-strand conformation polymorphism analysis, *Loss* heterozygous deletion, *Deletion* homozygous deletion

^¥^Gene symbols correspond to the NCBI Refseq names

**Fig. 4:**
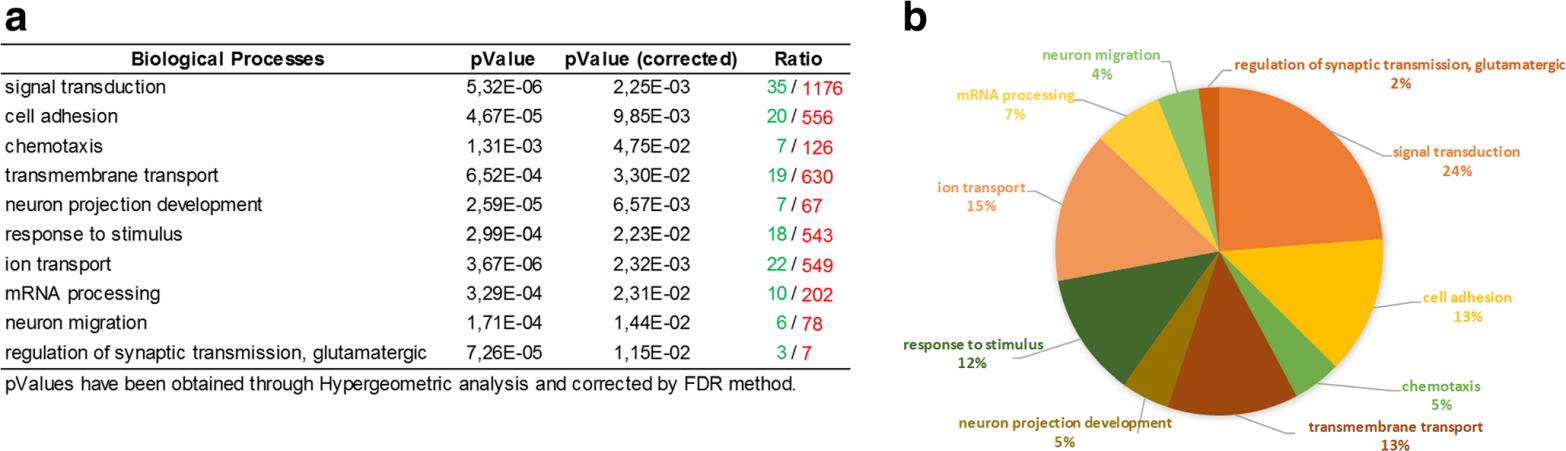
A representative illustration showing the functional correlation between ALS-associated CNV-affected genes and their biological processes. Interaction map represents the most promising candidate genes overlapping CNVs that have been consistently associated with ALS, grouped on the basis of the main biological processes associated with them. The map was created using the MetaCore Pathway Map Creator tool (GeneGo). Genes associated with CNV gain regions are labeled with *red dots* while genes associated with homozygous or heterozygous deleted CNVs are labeled with *blue dots*. The *“checkerboard” color* indicates genes displayed both CNV gains and losses. Detailed information about genes depicted in the figure and related biological processes are reported in Table 2. A detailed legend for the network objects is shown in Supplementary Fig. 1

In the following paragraphs, we review the most plausible candidate CNV loci that have been consistently associated with ALS, highlighting their potential functional impact in the pathophysiology of the disease (Tables 1 and 2 and Fig. 4).

**Table 2:** Genes overlapped with CNV loci with a potential relevance for ALS susceptibility

Gene	Description	Chromosomal location	GO Processes Associated	Type of CNV
SMN1 and SMN2	Survival motor neurons 1 and 2	5q13.2	mRNA processing, spliceosomal complex assembly, synaptic transmission	Gain and loss
NEFH	Neurofilament heavy subunit	22q12.2	Cellular response to oxidative stress, neurofilament cytoskeleton organization	Deletion
IDI1 and IDI2	Isopentenyl diphosphate delta isomerase 1 and 2	10p15.3	Cholesterol biosynthesis	Gain
DPP6	Dipeptidyl-peptidase 6	7q36.2	Regulation of neuronal action potential, regulation of potassium ion transport, protein localization to plasma membrane, proteolysis	Gain and loss
NIPA1	Non-imprinted in Prader-Willi/Angelman syndrome 1	15q11.2	Transmembrane transport	Loss
EPHA3	Ephrin type-A receptor 3	3p11.2	Axon guidance, cell adhesion, cell migration, signaling, cytoskeleton organization	Loss
AATK	Apoptosis-associated tyrosine kinase	17q25.3	Brain development, regulation of axon extension, neuron apoptotic process	Gain
BAIAP2	BAI1-associated protein 2	17q25	G protein coupled receptor signaling pathway, neurotransmitter secretion	Gain
CHMP6	Charged multivesicular body protein 6	17q25.3	Endosomal transport, membrane organization, nucleus organization, protein transport	Gain
IGFALS	Insulin-like growth factor binding protein acid labile subunit	16p13.3	Cell adhesion, cellular protein metabolic process, signal transduction	Gain
CACNA1H	Calcium channel, voltage-dependent, T type, alpha 1H subunit	16p13.3	Axon guidance, calcium ion import, membrane depolarization during action potential, muscle contraction, regulation of membrane potential	Gain
MAPK8IP3	Mitogen-activated protein kinase 8 interacting protein 3	16p13.3	JNK cascade, axon guidance, neuron projection development, positive regulation of neuron differentiation, regulation of gene expression, vesicle-mediated transport	Gain
BAIAP3	BAI1-associated protein 3	16p13.3	G-protein coupled receptor signaling pathway, neurotransmitter secretion	Gain
UBE2I	Ubiquitin-conjugating enzyme E2I	16p13.3	DNA repair, cell cycle, cell division, cellular protein metabolic process, chromosome segregation, nucleotide-excision repair, post-translational protein modification, protein ubiquitination	Gain
SPSB3	splA/ryanodine receptor domain and SOCS box containing 3	16p13.3	Intracellular signal transduction, protein ubiquitination	Gain
KCNIP4	Kv channel interacting protein 4	4p15.32	Potassium ion transport	Deletion
KCNB2	Potassium channel, voltage gated Shab related subfamily B, member 2	8q13.2	Potassium ion transport, synaptic transmission	Gain
KCNQ5	Potassium channel, voltage gated KQT-like subfamily Q, member 5	6q14	Potassium ion transport, synaptic transmission	Loss
GSDMD	Gasdermin D	8q24.3	Cellular response to extracellular stimulus	Gain & Loss
GRIK1	Glutamate receptor, ionotropic, kainate 1	21q22.11	Central nervous system development, glutamate receptor signaling pathway, ion transport, membrane depolarization, regulation of synaptic transmission, regulation of synaptic plasticity	Loss
GRIK2	Glutamate receptor, ionotropic, kainate 2	6q16.3	Central nervous system development, glutamate receptor signaling pathway, ion transport, membrane depolarization, regulation of synaptic transmission, regulation of synaptic plasticity, regulation of neuron apoptotic process, regulation of JNK cascade	Gain
EEF1D	Eukaryotic translation elongation factor 1 delta (guanine nucleotide exchange protein)	8q24.3	Cellular protein metabolic process, gene expression, mRNA transcription, positive regulation of I-kappaB kinase/NF-kappaB signaling, regulation of cell death, signal transduction	Gain & Loss
ATXN1	Ataxin 1	6p23	RNA processing, excitatory postsynaptic potential, regulation of transcription, regulation of glial cell proliferation	Loss
ATXN3L	Ataxin 3-like	Xp22.2	Cellular response to misfolded protein, protein deubiquitination, regulation of transcription	Gain
SLC1A7	Solute carrier family 1 (glutamate transporter), member 7	1p32.3	L-glutamate transmembrane transport, neurotransmitter secretion, synaptic transmission	Gain
TRDN	Triadin	6q22.31	Cellular calcium ion homeostasis, cytoplasmic microtubule organization, endoplasmic reticulum membrane organization, muscle contraction, transmembrane transport	Gain
CPLX1	Complexin 1	4p16.3	Exocytosis, glutamate secretion, regulation of exocytosis, synaptic transmission, synaptic vesicle exocytosis, transport	Gain
ANKRD11	Ankyrin repeat domain 11	16q24.3	Multicellular organism growth, tissue homeostasis	Gain
PPP1R13B	Protein phosphatase 1 regulatory subunit 13B	14q32.33	Apoptotic process	Gain
FOXO3	Forkhead box O3	6q21	DNA damage response, apoptotic process, cell differentiation, cellular response to oxidative stress, epidermal growth factor receptor signaling pathway, immune response, regulation of neuron differentiation, transcription, tumor necrosis factor-mediated signaling pathway	Gain
HFE	Hemochromatosis	6p21.3	Antigen processing and presentation, immune response, ion transport, regulation of proteasomal ubiquitin-dependent protein catabolic process, regulation of receptor activity, positive regulation of gene expression, regulation of protein binding, transport	Gain
GGTLC2	Gamma-glutamyltransferase light chain 2	22q11.22	Glutathione metabolic process	Loss
ATG7	Autophagy related 7	3p25.3	Autophagy, cellular homeostasis, cellular protein modification process, cellular response to hyperoxia, central nervous system neuron axonogenesis, cerebral cortex development, membrane organization, mitochondrion organization, regulation of apoptotic process, protein transport, protein ubiquitination	Loss
ANXA5	Annexin A5	4q27	Calcium ion transmembrane transport, regulation of apoptotic process, signal transduction	Loss
GEMIN6	Gem nuclear organelle-associated protein 6	2p22.1	Gene expression, mRNA processing, mRNA splicing	Loss
MTMR7	Myotubularin-related protein 7	8p22	Dephosphorylation	Loss
ACYP2	Acylphosphatase 2, muscle type	2p16.2	Phosphate-containing compound metabolic process	Loss
ZFP14	ZFP14 zinc finger protein	19q13.12	Regulation of transcription	Loss
FMN2	Formin 2	1q43	Actin filament assembly, cellular response to DNA damage stimulus, cellular response to hypoxia, intracellular signal transduction, intracellular transport, multicellular organismal development, regulation of apoptotic process, protein transport, vesicle-mediated transport	Deletion

*Loss* heterozygous deletion; *Deletion* homozygous deletion

### Survival Motor Neuron

Among candidate genes that may modulate susceptibility and disease course in ALS, a great deal of attention has been given to the survival motor neuron (*SMN*) gene. It maps in a highly duplicated region of chromosome 5 (5q13.3) and is present in two homologous copies: a telomeric copy called *SMN1* and a centromeric copy called *SMN2* that produces approximated only 20% of full-length transcripts coding for SMN [39]. This is a housekeeping protein that is essential for the efficient assembly and regeneration of spliceosomal small nuclear ribonucleoproteins (snRNP) in various human cell types, including the axons of spinal motor neurons, exerting important effects in protein translation, as well as in the mitochondria and cytoskeleton maturation and synaptic transmission regulation (Table 2 and Fig. 4) [40].

Aberrant CNVs in *SMNs* result in a defective protein, leading to deficits in snRNP assembly, disruption of normal cellular RNA metabolism, and motor neuron degeneration [41]. Homozygous deletions of *SMN1* are responsible for the pathogenesis of spinal muscular atrophy (SMA), an autosomal recessive motor neuron disease characterized by lower motor neuron loss and muscle atrophy, while *SMN2* copy numbers seem to modulate the severity of the SMA phenotype and survival [42].

Because of the phenotypic similarities between SMA and ALS, various studies have focused on the possible association between copy number abnormalities in *SMN* genes and ALS, but controversial results have been obtained (Table 1). Indeed, although several studies have demonstrated that high or low *SMN1* copy number together with the absence of *SMN2* increased risk of developing ALS [43–48], others have not found any significant association [49–51]. These apparently contradictory data, at least in part, may be due to the use of inadequate size populations and different assays used to assess CNVs in the *SMN* genes. Similar conflicting results were also observed at the SMN protein level. Indeed, both low and high levels of SMN production seem related to the risk of ALS. Upregulated expression of *SMN* genes was recently found in the motor cortex of SALS patients, supporting the evidence that overexpression of SMN protein may be toxic for motor neurons [52, 53]. Decreased levels of SMN result, instead, in a reduction of its chaperone-like activity against mutant SOD1-mediated toxicity in vitro, contributing to increase-free radical injury and oxidative stress, two well-established features of ALS [54]. In support of this theory, it has been demonstrated that an increase in the levels of SMN improved motor functions and delayed motor neuron loss in different cellular and animal models of ALS [55, 56].

Further evidence for the contribution of SMN signaling in ALS pathogenesis is based on the observation that SMN and some ALS-associated proteins share common biochemical pathways. This is the case, for example, of *FUS* and *TDP-43*, two gene-encoding proteins extensively associated with both sporadic and familial ALS that colocalize with SMN in subnuclear organelles known as gems (gemini of Cajal bodies). ALS-related mutations in these genes seem to contribute to motor neuron dysfunctions also by reducing the localization of SMN to axons with the consequent disruption of the SMN-mediated assembly and integrity of the splicing machinery [57–59].

Considering its potential impact on ALS pathogenesis, several studies have investigated the role of SMN as a pharmacological target in ALS. To this regard, the systemic administration of valproate (VPA), a drug able to increase the SMN levels in the SNC through an epigenetic mechanism, has shown neuroprotective effects both on SMA and ALS patients [60].

In addition to alterations in the activity of SMN complex, aberrant copy numbers in the *SMN* genes might confer susceptibility for ALS also through SMN-independent mechanisms, acting, for example, as markers of linkage disequilibrium with other ALS susceptibility loci, such as the neuronal apoptosis inhibitory protein (*NAIP*) gene. A more detailed and accurate molecular genetic investigation of the *SMN* gene region could reveal additional genetic variants with potential relevance for ALS, providing new insights into the common pathogenic mechanisms underlying ALS and other motor neuron diseases [61].

### Neurofilament Heavy Subunit

Neurofilaments (NFs) are the most prominent cytoskeletal components in neurons. They are composed of a globular head, α-helical rod region, and a globular tail and are expressed under three different neuron-specific subunits with different molecular weights: light (NEFL), medium (NEFM), and heavy (NEFH). NFs, together with microtubules and microfilaments, form part of the slow axonal transport and contribute to the formation and maintenance of the neuronal structure (Table 2 and Fig. 4) [62].

Although the role of NFs in ALS is not yet fully clarified, dysregulated expression and point mutations of *NFs* have been reported in human and animal models of ALS [52, 63, 64]. The principal pathogenic mechanism of these variations seems to be related to defects of the normal NFs assembling and phosphorylation. These alterations, in addition to dysfunction of the ubiquitine-proteasome system, may cause changes in the cross-linking properties of NFs, resulting in the aberrant accumulation of these proteins in motor neurons, which represents an established pathologic hallmark of ALS [65, 66].

Several studies have investigated the role of *NF* structural chromosomal alterations in ALS (Table 1). Deletions in *NEFL*, accompanied by a concomitant altered expression of *NEFH* and *NEFM* in the cell bodies and axons of motor neurons, have been associated with a significant delay of the disease onset and progression in ALS animal models [67]. In addition, point mutations (C2232T and C2414A) were also found in the short allele of *NEFH*, suggesting that these may act as markers for deletion mutants [68].

Significant associations were also established between the sporadic form of ALS and deletions in the C-terminal tail of NEFH [68]. This region is composed of a unique functional domain consisting of 43–45 repeat motifs of the amino acids lysine-serine-proline (KSP) [69]. The phosphorylation of these motifs in the C-terminal tail allows NEFH to interact with other cytoskeletal components (e.g., microtubule-associated proteins), regulating the interfilament spacing and thereby the axonal caliber. Deletions of the NEFH KSP domain have been detected in SALS patients as well as in some patients with a FALS pedigree, even if it was not yet determined whether the variant segregates with the disease or not [70, 71]. Interestingly, loss of a KSP motif, or multiples of this, seems to affect a recognition sequence for the neurofilament kinase CDK5, suggesting that deletions at this level may result in aberrant interactions of NEFH with other cytoskeletal elements, leading to axonal and cytoskeleton integrity destruction and motor neuron degeneration.

### Isopentenyl Diphosphate Isomerase

Although the exact association between alterations in lipid metabolism and ALS remains unknown, there are several evidence of possible involvement of cholesterol and other lipids in the disease pathogenesis. Decreased cholesterol levels as well as dysfunctions in lipids transport and metabolism have been found in both human and ALS animal models while increasing dietary lipid content appears to be associated with neuroprotective effects [72–75].

Among proteins involved in the lipid metabolism is the isopentenyl diphosphate isomerase (IDI), a cytoplasmic enzyme belonging to the mevalonate pathway that plays an essential role in the biosynthesis of cholesterol and other lipophilic molecules (Table 2 and Fig. 4). IDI has two isoforms in humans, IDI1 and IDI2 that are encoded by two tandemly duplicated genes [76, 77].

Several studies support the role the mevalonate pathway and IDIs in the pathogenesis of sporadic ALS. Downregulated expression of *IDI1* was recently found in the motor cortex of a specific subgroup of SALS patients [52]. Moreover, a segmental copy number gain, encompassing the *IDI1*/*IDI2* gene region on 10p15.3 subtelomere, was found in SALS patients. This duplication leads to the disruption of the genetic composition and reduction of the *IDI1* expression (Table 1) [78]. The association between *IDI1/IDI2* copy number alterations and the sporadic form of ALS may be explained by the instability of this genomic region that contains multiple low-copy repeats in a small region of the genome. These segmental duplications can arise from an unequal crossing-over or end-joining events, suggesting the possibility that de novo CNVs may occur [79]. Further investigation about the function of IDI1/IDI2 in motor neurons may allow to obtain new insights into the pathogenesis of SALS and to identify also novel promising therapeutic targets for this disease.

### Dipeptidyl-Peptidase 6

Dipeptidyl aminopeptidase-like protein 6 (DPP6) is a member of the prolyl oligopeptidase family of serine proteases. It is predominantly expressed in the central nervous system which modulates the function and expression of potassium channels as well as excitability at the glutamatergic synapse (Table 2 and Fig. 4).

Although the role of *DPP6* in ALS pathogenesis remains unclear, genetic variations in *DPP6* have been identified as potential risk factors for different human diseases, including ALS [6, 80–82]. Several genome-wide studies, in fact, have reported an association between an SNP (rs10260404) in *DPP6* and ALS susceptibility and increased expression of *DPP6* was found in motor cortex of SALS patients [6, 52, 83]. In addition, deletions and duplications affecting *DPP6* were also found in some cases of ALS (Table 1) [84]. These copy number aberrations occur mainly at the 5′ end of the gene, a known CNV region flanked by segmental duplications mediating genomic rearrangements [85]. The functional impact of these genomic aberrations seems to involve the generation of altered or truncated transcripts as well as the disruption of regulatory elements in this region that may alter gene expression. Further investigations are necessary to better clarify the function of DPP6 in the nervous system and its potential role in ALS susceptibility.

### Non-imprinted in Prader-Willi/Angelman Syndrome

Non-imprinted in Prader–Willi/Angelman syndrome (NIPA1, also known as SPG6) is an inhibitor of bone morphogenic protein signaling, one pathway that plays an important role in the formation, maintenance, and function of synapses as well as in transmembrane transport (Table 2 and Fig. 4). Despite mutations in *NIPA1* not being directly linked to ALS pathogenesis, they are known to cause hereditary spastic paraplegia (HSP) type 6, a neurodegenerative disease characterized by the selective degeneration of upper motor neurons [86]. Although ALS and HSP are clinically different, they share important clinical features, including selective motor neuron degeneration. Mutations in some genes causing various forms of HSP, such as *SPAST* and *SPG11*, were also associated with ALS phenotype [87]. In particular, *SPG11* encodes spatacsin, another inhibitor of bone morphogenic protein signaling, supporting the involvement of this pathway in motor neuron degeneration and pathogenesis of both ALS and HSP.

NIPA1 polyalanine repeat expansions have been identified as potential risk factors for ALS and appear involved in the modulation of the disease course. Moreover, several CNVs affecting *NIPA1* were also found in SALS patients (Table 1) [84, 88]. In particular, these copy number alterations affect a known CNV region of chromosome 15 (15q11.2) flanked by segmental duplications. While duplications in this region do not seem to contribute to ALS susceptibility, deletions overlapping four adjacent genes, including *NIPA1*, showed a strong statistical association (Table 1) [84]. Recently, CNVs in the *NIPA1* region have been also associated with other neurological diseases, including schizophrenia and epilepsy, suggesting that *NIPA1* and other genes inside the same CNV-driven region, in association with other risk factors, may concur to cause these different pathologic phenotypes [89].

### Ephrin Type-A Receptor 3

Ephrin receptors (Ephs) represent the largest known subfamily of tyrosine kinase receptors. Ephrin receptors and their ligands are involved in a variety of important functions, including axonal outgrowth, cytoskeletal structure development, neuronal connectivity, neuronal apoptosis, synaptic maturation, and plasticity (Table 2 and Fig. 4) [90, 91].

Alterations in the expression or function of ephrins and their receptors induce pathological changes in the motor neuron circuitry, contributing to the initiation and progression of ALS pathogenesis [92]. In accordance with this theory, deregulated expression of ephrins and their receptors was found in the motor cortex and spinal cord of SALS patients [3, 93] and several SNPs affecting Eph/ephrin genes have been associated with ALS susceptibility [94]. It is of interest to note that some of these alterations often result in beneficial effects. Indeed, loss-of-function mutations in *EPHA4* or complete knockdown of this gene have shown to rescue and prolong survival in ALS animal models [95]. In addition, a heterozygous deletion of *EPHA3* was found significantly higher in controls as compared to ALS patients, supporting a potential protective role of this variant against the risk of developing ALS (Table 1) [96]. All these evidence support the importance of Eph/ephrin signaling in modulating the vulnerability of motor neurons to axonal degeneration, highlighting the necessity to further investigate this protein family as promising therapeutic candidates for ALS.

### Mitochondrial DNA Genes

Impaired mitochondrial bioenergetics function plays an important role in several neurodegenerative diseases, including ALS. In particular, dysfunctions in mitochondria result in a reduction in bioenergetics efficiency, increasing the risk of neuronal death when energy demands exceed cellular energy production [97, 98].

One of the genes primarily involved in oxidative phosphorylation encodes cytochrome c oxidase (CO), the terminal component of the mitochondrial respiratory chain that transfers electrons from reduced cytochrome c to molecular oxygen. CO is a multiheteromeric enzyme composed of 13 protein subunits, some of which coded by mitochondrial DNA (mtDNA) genes [99, 100]. Reduced levels of CO histochemical activity as well as mutations and heterozygous deletions in mtDNA genes encoding some CO subunits (such as *CO3* and *COX7C*) have been detected in patients with ALS phenotype, contributing to reduce bioenergetic functions observed in ALS motor neurons (Fig. 4) [101–104].

In addition to genetic variations of mtDNA CO genes, deletions in other mtDNA genes were found in substantia nigra neurons, where it seems to cause cellular respiration deficits, reduced capacity to modulate synaptic activity, and swings in cytosolic calcium levels as well as reduced capacity to survive to excitotoxic stresses [105, 106]. Although it is not yet fully clarified how these mtDNA deletions arise, their origin may involve failures in the mitochondrial biogenesis programs as well as genetic, epigenetic, or post-translational causes (or their combinations) [107, 108]. Similar chromosomal rearrangements have been also observed in ALS phenotype. Indeed, high abundances of deletions involving *ND2* and *ND4*, two mtDNA genes that code for NADH-ubiquinone oxidoreductase, an essential respiratory protein complex, were found in the spinal cord of ALS patients, supporting the concept that some motor neurons in ALS may die because of a deficit in the mitochondrial ATP production and cellular energy metabolism (Fig. 4) [102].

It is interesting to note that while some ALS patients were characterized by an accumulation of mtDNA deletions, other ALS cases showed abundant mtDNA copy numbers and relatively low levels of deletions. This observation is corroborated by our previous work that described a coordinated decrease of several gene-encoding proteins involved in the oxidative phosphorylation pathway only in a specific subgroup of SALS patients, sustaining the existence of a molecular heterogeneity in ALS patients [52]. Based on these results, it is evident that mitochondria-targeted therapies, aimed to provide and/or express intact mtDNA in motor neurons, may represent a helpful strategy for the management of bioenergetics defects in specific subgroups of ALS patients [109].

### Multiple Rare CNVs: Assessing their Role through a System Biology Approach

Despite their importance in deciphering the genetic cause underlying ALS, common CNVs account for only a relatively small fraction of the genetic variation, while the contribution of less frequent but potentially functionally important variants is not yet fully elucidated.

Recent advances in high-throughput genomic technologies enabled the identification of a large number of rare and novel ALS-specific CNV loci, which were found in ALS patients but absent or extremely rare in individual controls of each study and/or in >2500 controls present in DGV (Table 1 and Supplementary Table 1) [110, 111]. However, no significant association was found between these rare variants and the risk of developing ALS. This may be mainly due to the effect size of most of these variants that is too small to be detected by using the traditional statistical methods. In addition, currently, the majority of genome-wide CNV studies typically apply a single-gene or single-variant approach, focusing their attention on the identification of a restricted list of potential candidates, previously implicated in the disease, without taking into account the potential joint impact of these rare variants in the etiopathogenesis of ALS [112]. To this regard, the new *systems biology* perspective may represent a powerful tool to overcome these limitations, enabling to characterize, in a comprehensive or high-throughput manner, the collective effects of these variants that, both individually or in combination, can contribute to increase the likelihood of developing ALS (Fig. 2).

**Fig. 5:**
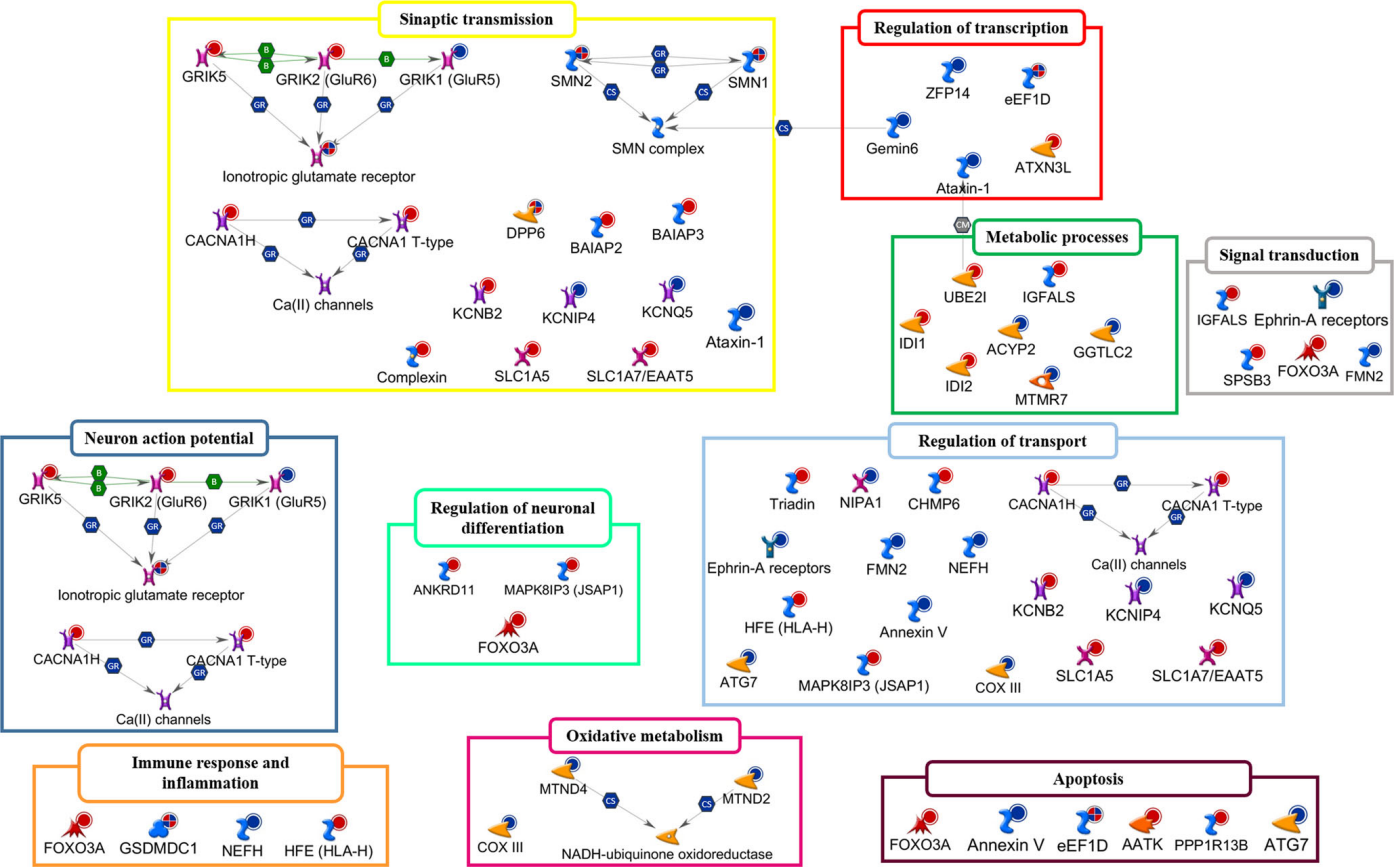
The functional enrichment analysis of the most plausible candidate genes overlapping ALS-specific CNV loci reveals biological processes relevant to ALS pathogenesis. **a** Representation of the top 10 most significantly enriched (*p* value <0.05) canonical GO biological processes associated with genes significantly enriched in rare and novel ALS-specific copy number changes (not reported in controls of each of the individual studies and/or in >2500 controls present in DGV). The analysis was performed using the Gene Ontology and KEGG databases and the list is arranged in descending order with the most significant GO biological processes at the *top*. Detailed information about the entire list of genes affected by ALS-specific CNV loci are reported in Supplementary Table 1. **b** GO term pie chart of the top 10 enriched (*p* < 0.05) “Biological processes” for genes overlapping ALS-specific CNV loci. GO terms or biological features of candidate CNV-affected genes and the percentage of genes represented in each category are indicated

**Fig. 6:**
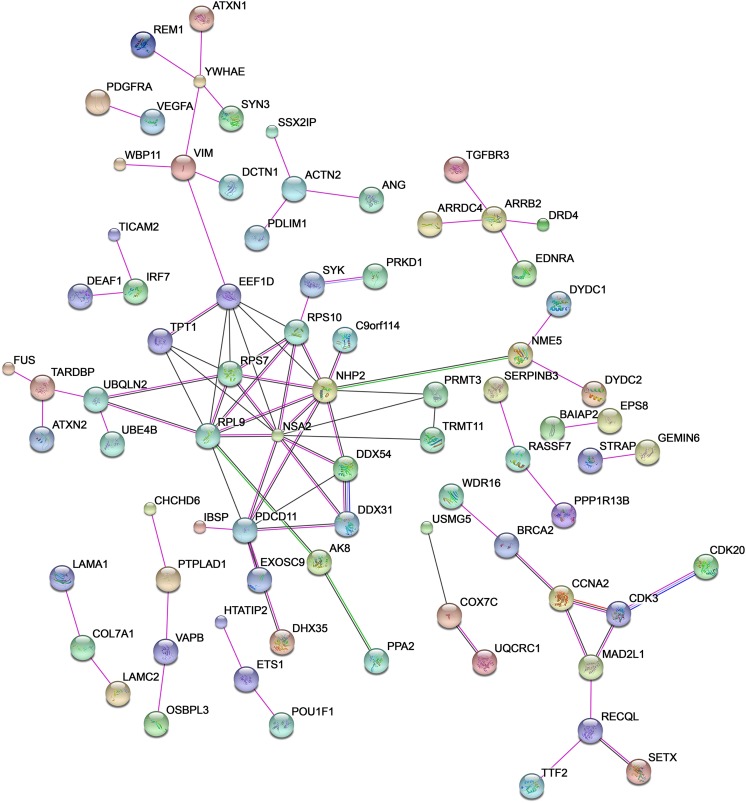
Functional network of known and predicted interactions between proteins encoded by genes affected by rare ALS-specific CNV loci and the most known causative ALS genes. The network was produced by the Search Tool for the Retrieval of Interacting Genes/Proteins (STRING) v10 (http://string-db.org/) using default settings. Proteins are represented by *spheres*. *Lines* linking proteins indicate evidence for interactions: a *red line* indicates the presence of gene fusion (genes that are sometimes fused into single open reading frames); a *green line* gene neighborhood (genes that reside within 300 bp on the same strand in the genome); a *blue line* co-occurrence (gene families whose occurrence patterns across genomes show similarities); a *purple line* experimental evidence (interaction extracted from protein-protein interaction databases); a *yellow line* text mining (interaction extracted from scientific literature); a *light blue line* database (interaction extracted from curated databases); a *black line* co-expression (proteins whose genes are co-expressed in the same or in other species)

Investigating the potential functional role of rare ALS-related CNVs, it emerges that, albeit these variants are individually rare, the target set of genes and related products may be annotated to one or more common biochemical pathways relevant to ALS pathogenesis, including signal transduction (*p* value = 5.32 E^−06^), cell adhesion (*p* value = 4.67 E^−05^), ion transport (*p* value = 3.67 E^−06^), and messenger RNA (mRNA) processing (*p* value = 3.29 E^−04^) (Table 2 and Figs. 4 and 5). Moreover, many of these genes show a functional correlation also with the most known causative ALS genes (e.g., *SOD1*, *ALS2*, *SETX*, *FUS*, *TARDBP*), supporting the evidence that multiple common and rare CNVs may exert their pathogenic effect by different multifactorial combinations, jointly contributing to the genetic susceptibility of ALS (Fig. 6). Interestingly, some of the genes affected by rare CNVs encode proteins that are differentially expressed in motor cortex of SALS patients, sustaining the theory that the genomic structural variants frequently affect the transcriptional regulation [52]. Below, we provide a brief description of the most plausible candidate genes affected by rare ALS-related CNVs in the light of the main biological processes in which are involved, in order to investigate their contribution in ALS pathogenesis and their relevance as potential diagnostic biomarkers (Fig. 4, Tables 1 and 2 and Supplementary Table 1).

#### Regulation of Synaptic Transmission and Neuronal Action Potential

Neurons are physically connected to each other to form extensive neural networks; the synaptic transmission is the process by which information rapidly flows in neuronal networks. This is essential for maintaining normal brain function as well as muscle tone and muscle movements coordination [113]. The disruption of the synaptic stability result in a disruption of neuronal circuits that represents almost certainly one of the major underlying causes of many neurologic diseases, including ALS, Parkinson’s and Alzheimer’s disease [114–116].

Multiple GWAS studies have described ALS-specific gains and losses in several genes encoding proteins involved in synaptic transmission and the regulation of neuronal action potential (*KCNIP4*, *KCNB2*, *KCNQ5*, *GRIK1*, *GRIK2*, *ATXN1*, *BAIAP2*, *CPLX1*, *CACNA1H*, *SLC1A7*) (Tables 1 and 2 and Supplementary Table 1) [110, 117].

Different CNVs in some genes that code for potassium and calcium channels (*KCNIP4*, *KCNB2*, *KCNQ5*, *CACNA1H*) were found in ALS patients and seem to have a particular interest in the deciphering the genetic cause of ALS (Tables 1 and 2 and Fig. 4). In fact, alterations in the homeostasis and signaling pathways of these ions seem to be responsible for some important toxic pathways underlying ALS, such as oxidative stress, mitochondrial dysfunction, and neuroinflammation [118]. In particular, an autoimmune attack on potassium channels has been identified as a possible risk factor for ALS and alterations in other potassium channel-related genes (e.g., *DPP6*) were found in several cases of ALS [119]. Moreover, deregulated expression of multiple genes encoding calcium and potassium channels were also found in motor cortex of SALS patients [52].

Changes in copy number and in the expression levels of genes encoding glutamate ionotropic receptors (*GRIK1*, *GRIK2*) were identified in several cases of ALS (Table 2 and Fig. 4) [52, 120]. Glutamate receptors mediate fast excitatory synaptic transmission in the central nervous system and their excessive stimulation as well as deregulations in their expression and activity are responsible for excitotoxicity, a phenomenon that has been regarded as one firm mechanism implicated in both acute and chronic neurodegenerative diseases, including ALS [120]. A copy number gain in *SLC1A7* (also known as *EAAT5*), a gene encoding a member of the glutamate transporter family, was found in a case of ALS but not in controls or DGV. Altered expression of this gene was found in the brains of patients with both familial and sporadic forms of ALS, supporting the role of an aberrant glutamate signaling in ALS pathogenesis (Table 2, Fig. 4, and Supplementary Table 1) [121].

Deletions in *ATXN1* and a duplication of the related gene *ATXN3L* were identified in some ALS patients (Supplementary Table 1) [110]. *ATXN1* encodes a protein mainly implicated in mRNA processing (Table 2 and Fig. 4) that normally contains a polyglutamine (polyQ) tract with 22–23 repeats; the occurrence of an expanded polyQ repeat has been associated with an increased genetic risk for ALS [122]. In addition to structural aberration, decreased expression of *ATXN1* was recently found in a specific subgroup of SALS patients [52]. ATXN1 seems also to be involved in transcriptional repression of angiogenic factors whose deregulations were widely associated with ALS pathogenesis [123]. Another gene involved in the regulation of angiogenic signaling pathway is *BAIAP2*, a gene encoding a brain-specific angiogenesis inhibitor that is able to modulate synaptic transmission as well as learning and memory (Table 2 and Fig. 4) [124]. Segmental copy number gains in *BAIAP2* and other structurally related genes (e.g., *BAIAP3*), were found in some cases of ALS patients (Table 1) [110, 111]. In addition, increased expression of *BAIAP2* and *BAIAP3* was found in motor cortex of SALS patients, supporting the theory that an excessive angiogenesis may contribute to ALS pathogenesis.

Copy number gains in *CPLX1* have been identified at a significantly higher frequency in ALS patients compared to controls (Table 1) [110]. *CPLX1* encodes a protein involved in synaptic vesicle exocytosis whose deregulation has been implicated in the pathogenesis of several neurological and neurodegenerative diseases, including ALS (Table 2 and Fig. 4) [52, 125].

#### Transcriptional Regulation

Mounting evidence has emerged to support an important role of transcription dysregulation in the initiation and progression of several neurodegenerative diseases, including ALS. Indeed, widespread aberrations in the molecular machinery that regulate gene expression and mRNA splicing have been reported both in sporadic and familial forms of ALS [126, 127]. Although the mechanisms and causal relationships have not yet been fully elucidated, several genome-wide studies have identified ALS-linked mutations and ALS-specific copy number variations in genes involved in the regulation of RNA processing and, thus, in the control of gene expression. In particular, the deletion of an intron of *ZFP14* was found in two ALS patients but not in individual controls (Table 1) [111]. The functional group of ZFP14 proteins exerts diverse functions, many of which have been extensively implicated in ALS, such as RNA packaging, transcriptional activation, regulation of apoptosis, as well as protein folding and assembly (Table 2 and Fig. 4).

A large deletion affecting *GEMIN6*, another gene involved in the control of neuronal gene expression, was found in a single ALS patient (Supplementary Table 1). GEMIN6 is mainly implicated in the regulation of mRNA processing and belongs to the SMN complex, which as previously discussed is a potential risk factor in ALS susceptibility (Table 2 and Fig. 4). In particular, a reduced expression of *GEMIN6* has been associated with a reduced activity of the SMN complex, contributing to alter normal snRNP assembly and leading to motor neuron degeneration [128].

A process that has been directly implicated in genetic causes of various motor neuron diseases is the enzymatic delivery of aminoacyl transfer RNAs (tRNAs) to the ribosome. Among genes involved in this process is *EEF1D*, a gene encoding a subunit of the elongation factor-1 complex. Copy number gains and losses in *EEF1D* were found in a modest number of ALS patients and increased expression of *EEF1D* was recently found in SALS patients, confirming the potential role of this factor in ALS pathogenesis (Tables 1 and 2 and Fig. 4) [52, 110].

#### Immune Response and Inflammation

Immune processes inside the central nervous system contribute to the maintenance of homeostasis and play important roles in resolving inflammation and mediating neuroprotection and repair [129]. Immune responses are finely regulated by multiple checkpoints that are responsible to ensure the protection of neuronal tissue from harmful events.

There is strong evidence that the acute neuroinflammation and the dysregulated immune response are potentially pathogenic factors in a number of neurodegenerative diseases, including ALS [130]. To this regard, aberrant gene expression and copy number variations in genes involved in neuroinflammation and cellular response to injurious stimuli (*FOXO3*, *HFE*, *GSDMD*) have been identified in ALS patients with respect to controls (Table 1 and Supplementary Table 1) [99, 110, 117]. In particular, *FOXO3* encodes a transcription factor involved in apoptosis, cellular metabolism, and resistance to oxidative stress, while variations in *HFE*, a gene encoding a protein involved in antigen presentation and processing, have been reported to significantly increase the risk of SALS in a number of different individuals (Table 2 and Fig. 4) [52, 131, 132]. ALS-specific copy number gains and losses were found also in a genomic region encompassing *GSDMD*, a gene encoding a member of the gasdermin family that appears to play a role in neuroinflammatory and caspase-induced apoptotic processes (Tables 1 and 2 and Fig. 4).

#### Signal Transduction

Signal transduction refers to the process through which an extracellular biological molecule, by interating with specific cell-surface receptors, activates a biochemical chain of events inside the cell, eventually eliciting a response. Neuron-specific alterations in various signal transduction pathways represent one of the principal pathological hallmarks of several neurological diseases, including ALS [133, 134]. An elevated number of genes encoding several trophic factors and their receptors as well as proteins involved in the regulation of intracellular signaling cascades were found differentially expressed in SALS motor cortex [52]. In addition, rare copy number aberrations in genes involved in multiple signaling cascades (*IGFALS*, *SPSB3*, *FOXO3* and *FMN2*) were also found in some cases of ALS, suggesting that alterations in these genes, either alone or in combination, may concur to generate motor neuron injury in ALS.

*IGFALS* encodes a serum protein that binds insulin-like growth factors (IGFs), increasing their half-life and their vascular localization. Increased expression of genes encoding IGF receptors and IGFALS was found in motor cortex of SALS patients [52]. In addition, structural gains in the genomic region encompassing *IGFALS* gene were identified in some ALS cases, supporting the hypothesis that dysregulation of this signaling cascade may be critical for ALS pathogenesis (Tables 1 and 2 and Fig. 4) [110, 135]. It has been also observed that pharmacological inhibition of the IGF signaling pathway exerts neuroprotective effects in different models of neurodegeneration [136]. In addition to *IGFALS*, another gene involved in the regulation of the intracellular signal transduction maps within the region of gain at chromosome 16 (Tables 1 and 2 and Fig. 4) [110]. This is *SPSB3*, a gene encoding a protein involved in the ubiquitine-proteasome pathway, the major proteolytic quality control system in cells, whose deregulation has been associated with numerous neurodegenerative diseases, like ALS, leading to abnormal protein aggregation and consequent neuron death [137].

Several GWAS studies have confirmed the presence of a homozygous deletion affecting an intron of *FMN2* gene in some cases of ALS but not in controls (Table 1) [80, 110, 138]. FMN2 is expressed in brain and spinal cord and plays an important role in the regulation of cytoskeletal assembly, signal transduction, and protein transport (Table 2 and Fig. 4). Decreased expression of this gene has been related to age-dependent memory loss in mice and SNPs in this gene have been detected in SALS patients [139, 140].

#### Metabolic Homeostasis

Maintaining a metabolic homoeostasis requires a balance between energy intake and expenditure. Growing evidence highlights that an imbalance in energy metabolism may contribute to the selective death of neurons in ALS [141]. To this regard, copy number abnormalities in some genes involved in metabolic processes (*IGFALS*, *UBE2I*, *ACYP2*, *MTMR7*) have shown to be potential risk factors for ALS (Table 1 and Supplementary Table 1). In particular, a structural gain on chromosome 16 encompassing multiple genes potentially involved in ALS, including *UBE2I*, was found in a moderate number of ALS patients (Table 1) [110]. *UBE2I* encodes an ubiquitin-conjugating enzyme involved in the ubiquitin-proteasome system that is crucial to a vast array of cellular processes, such as cell cycle control, immune responses, and metabolic regulation [142]. Recently, an upregulated expression of UBE2I was identified in astrocytes from ALS animal models and a differentially expression of its gene was also found in motor cortex of SALS patients [52, 143, 144].

Heterozygous deletions of *MTMR7* and *ACYP2* genes were found in a limited number of ALS patients, but not in controls or DGV (Supplementary Table 1) [84, 96, 145]. MTMR7 is expressed specifically in the brain and is involved in the lipid metabolism and phosphatidylinositol signaling pathway, a mechanism implicated in ALS pathogenesis (Table 2 and Fig. 4) [146]. *ACYP2* encodes a protein belonging to enzyme family that acts as a phosphatase and is implicated in metabolic processes and Ca^+2^ modulation (Table 2 and Fig. 4). Interestingly, deletions of *ACYP2* have been associated with a relatively early age of disease onset (32–44) in ALS patients, and its reduced expression has been found in the motor cortex of SALS patients [52].

Another interesting heterozygous deletion encompassing *GGTLC2* was found significantly higher in controls as compared to ALS patients, suggesting a potential protective role of this variant against the risk of developing ALS (Table 1). *GGTLC2*, whose decreased expression was found in SALS patients [52], encodes a protein involved in glutathione metabolism, a process associated with altered energy metabolism in ALS pathogenesis (Table 2 and Fig. 4) [147, 148].

#### Apoptosis

Apoptosis is a type of programmed cell death that plays an essential role during embryogenesis, cellular homeostasis, immune system maturation, and cellular response to external stimulus, including DNA damage and oxidative stress. There is strong evidence to suggest that apoptosis is responsible for motor neuron degeneration in ALS [149]. Apart from genetic mutations and changes in gene expression, copy number aberrations in genes encoding factors involved in the control of apoptosis were detected only in ALS patients and absent in controls, suggesting a possible contribution of these variants in increased risk of developing ALS (Table 1 and Supplementary Table 1). In particular, heterozygous deletions in regions encompassing *ATG7*, *ANXA5*, and *PPP1R13B* genes and a segmental gain in the *AATK* gene were observed (Table 1 and Supplementary Table 1) [111, 138, 145]. ATG7 is a crucial factor for the induction of autophagy and ubiquitin-related activities as well as in apoptosis and the maintenance of axonal homeostasis (Table 2 and Fig. 4) [150]. Interestingly, deletions of *ATG7* have been reported to induce axonal degeneration in a mouse model, leading to accumulation of polyubiquitinated aggregates and severe neurodegeneration. *ANXA5* encodes a protein that plays an essential role in apoptosis and survival as well as neurite growth of in vitro cortical neurons and has been previously indicated as a candidate gene for ALS (Table 2 and Fig. 4) [151, 152]. *PPP1R13B* encodes an apoptosis-stimulating protein that is able to interact with p53, regulating neuronal differentiation and specifically enhancing p53-induced apoptosis that was identified as an apoptotic mode of motor neuron cell death in the spinal cord of ALS patients (Table 2, Fig. 4, and Supplementary Table 1) [153–155]. *AATK* encodes a protein that plays an essential role in the induction of mature neuron apoptosis, neuronal genesis and differentiation, as well as axon outgrowth. Copy number gains in *AATK* are in accordance with the evidence that an increased expression of this gene is induced during apoptosis, supporting a role of this signaling pathway in ALS (Table 2 and Fig. 4) [156].

#### Neuronal Development and Differentiation

Neuronal development is a process strongly dependent from a balanced and tightly regulated control of several trophic factors capable of regulating important physiological processes, including neuronal survival, migration, and differentiation as well as synapse and dendrite maintenance and axonal outgrowth [157]. Alterations to crucial genes regulating neural development and differentiation can lead to impairments in the cellular homeostasis and have been associated with numerous neurodegenerative diseases, such as Huntington’s and ALS [158, 159]. In addition to changes in genetic and epigenetics factors, through mutation or altered gene expression, copy number variations were associated as potential risk factors for ALS also. In fact, segmental duplications in genes involved in the regulation of neuronal cell death and differentiation (*FOXO3*, *ANKRD11*, *MAPK8IP3*) were found in some cases of ALS (Table 1).

ANKRD11 is a large nuclear protein that regulates transcription, potentially by binding chromatin-modifying enzymes and is involved in apoptotic processes by enhancing the transcriptional activity of p53 proteins (Table 2, Fig. 4 and Supplementary Table 1) [153, 154]. A structural gain in an intron of *ANKRD11* was identified only in two ALS patients compared to control subjects [99] (Table 1). In addition to structural aberrations, mutations and altered expression of *ANKRD11* have been associated with perturbations in neural development occurring in some neurological conditions, such as autism [160].

*MAPK8IP3* encodes a protein that binds and regulates the activity of numerous protein kinase components of the c-Jun N-terminal kinase (JNK) signaling pathway, facilitating JNK activation and thus playing a role in the regulation of many cellular events, including growth control, apoptosis, and axonal transport [161]. Increased levels of MAPK8IP3 seem to be required for neurodegeneration in different models of neurological disorders, including Parkinson’s and Alzheimer’s diseases, suggesting that accumulations of these proteins may represent a response to oxidative stress conditions [162, 163].

#### Neuronal Transport

The trafficking and transport of vital cellular components is critical for correct neuronal function and axonal maintenance. Increasing evidence has highlighted a correlation between dysfunction of the cellular transport machinery, for example caused by an oxidative damage, and many neurodegenerative conditions, including ALS [164]. Besides point mutations in genes extensively related to ALS pathophysiology (e.g., *C9orf72*, *TDP-43*, *VAPB*, *DCTN1*), several copy number aberrations encompassing genes involved in the regulation of neuronal transport were found in ALS patients. In addition to some genes that were previously discussed in the above paragraphs (*ATG7*, *NIPA1*, *CACNA1H*, *KCNIP4*, *KCNQ5*, *KCNB2*, *SLC1A7*, *CO3*, *MAPK8IP3*, *ANXA5*, *HFE*, *FMN2*, *NEFH*, *EPHA3*), two interesting segmental duplications in *TRDN* and *CHMP6* were found in some cases of ALS and in none of patient controls (Supplementary Table 1) [84, 138, 165]. *TRDN* encodes an integral membrane protein highly expressed in skeletal muscle with a recognized role in calcium release, and whose low expression has been found in brain and nerve tissue, suggesting its potential role in motor neuron diseases (Table 2, Fig. 4, and Supplementary Table 1) [166]. *CHMP6* encodes a member of CHMP subfamilies involved in the formation of endocytic multivesicular bodies that are required for autophagic clearance of protein aggregates (Table 2 and Fig. 4). Deregulation in this process leads to abnormal ubiquitin-positive protein deposits in neurons that represent a hallmark of several neurodegenerative diseases, including ALS. In addition, mutations in another component of CHMP family (*CHMP2B*) have been widely implicated in frontotemporal dementia and ALS [167], sustaining the role of this protein family in ALS pathogenesis.

#### Rare ALS-Related CNVs in Intergenic Regions

In addition to CNVs overlapping with genes, several rare non-polymorphic structural variants overlapping with promoters or intergenic regions have been reported as potential ALS candidate CNVs. Although these regions are difficult to decode, the possibility of long-acting gene regulatory zones residing in these regions cannot yet be excluded. Among these, of particular interest are two heterozygous deletions affecting intergenic regions of chromosome 11, in the proximity of the centromere, and chromosome 19 (Table 1) [96]. Despite CNVs overlapping, telomeric and centromeric chromosome regions should be treated with more caution because of the lower probe density coverage that makes them more prone to false CNV calls than other regions of the genome, a significant association was found between CNVs in these regions and an increased risk of developing ALS (Table 1).

Several interesting CNV-driven genomic regions encompassing the promoter region of *RPS3A*, *DHX30*, *UGT8* and *CHL1* were also found in ALS patients (Table 1) [138]. *RPS3A* encodes a ribosomal protein that plays roles in regulating cell growth, transformation and death. *DHX30* encodes an RNA helicase that is thought to be an accessory subunit of the mitochondrial ribosome and whose depletion has been associated with alterations in the levels of mitochondrial mRNAs [168, 169]. UGT8 is a protein belonging to a glycosyltransferase family involved in lipid biosynthesis and metabolism, a process implicated in ALS pathogenesis. CHL1 is a neural cell adhesion molecule that plays a crucial role in axonal guidance and maintenance of neural circuits [170].

Other ALS-related CNVs were found in regions of chromosomes 5, 8, and 11 flanking *HCN1*, *POTEA*, *OR4A5*, and *OR4C12*, respectively (Table 1) [138]. *HCN1* encodes a major component of the hyperpolarization-activated cyclic nucleotide-gated channels that seems to enhance hippocampal-dependent learning and memory and modulate synaptic transmission and plasticity [171]. Duplications in *POTEA* have been already reported in some cases of early onset Alzheimer’s disease [172]. Olfactory receptor genes (e.g., *OR4A5* and *OR4C12*) have been directly or indirectly involved in neuronal injury and nerve regeneration, and their dysregulation was associated with several neurodegenerative conditions, like Alzheimer’s and Parkinson’s diseases [173, 174].

## Conclusions

Despite intensive researches and incredible technological advancements, ALS still remains a fatal incurable neurodegenerative disease in which the majority of cases are diagnosed in advanced stages with limited treatment options and poor prognosis. The most important challenges in the clinical management of ALS are the better understanding of causes and mechanisms underlying motor neuron degeneration as well as the identification of precise diagnostic biomarkers and effective therapeutic strategies.

To achieve a comprehensive understanding of the complex genetic-environmental interactions underlying ALS susceptibility, all forms of genetic variation need to be addressed. In addition to the contribution of SNPs, which account for only a limited number of familial and sporadic ALS cases, evidence suggests that other genomic variants, such as CNVs, appear to have a more dramatic impact on human disease phenotype and represent important clues for the deciphering genetic susceptibility to complex diseases, like ALS. However, the traditional single-gene analysis generally accounts for only a small proportion of the phenotypic variation in ALS while is often inadequate for evaluating the collective effects of multiple rare CNVs on disease risk.

The systems biology paradigm represents an innovative way for analyzing the complex underlying biological processes, providing new instruments to fill interstices of the intricate mosaic of ALS pathogenesis and generate a more definite molecular picture of this disease.

Here, we provided some insights into the associations between common and rare CNVs and ALS pathogenesis, focusing on the characterization of the potential CNVs’ impact on different signaling pathways whose deregulation seems to contribute to motor neuron degeneration in ALS. Overall, the use of this integrative genomics approach promises to enlarge our knowledge about genetic and molecular risk factors for ALS and, even in the light of the molecular heterogeneity emerged from our previous work [52], offers an important stepping-stone for the understanding, diagnosis and treatment of complex and multifactorial diseases, like ALS.

We believe that future comprehensive analyses of structural genomic variations together with other high-resolution genotyping data, could help to provide a better definition of the molecular signatures of ALS, laying the basis for a more accurate and precise molecular biomarker-assisted diagnosis and the ultimate development of more effective and personalized therapeutic strategies (Fig. 2).

Cu/Zn superoxide-dismutase gene (SOD1); alsin (ALS2); senataxin (SETX); spastic paraplegia type 11 (SPG11); FUS RNA binding protein (FUS); vesicle-associated protein B (VAPB); angiogenin (ANG); TAR DNA binding protein (TARDBP); FIG4 phosphoinositide 5-phosphatas (FIG4); optineurin (OPTN); ataxin 2 (ATXN2); ubiquilin 2 (UBQLN2); granulin (PGRN); profilin 1 (PFN1); dynactin 1 (DCTN1); chromosome 9 open reading frame 72 (C9ORF72); vascular endothelial growth factor A (VEGFA); FGGY carbohydrate kinase domain containing (FGGY); dipeptidyl-peptidase like 6 (DPP6); inositol 1,4,5-trisphosphate receptor type 2 (ITPR2); kinesin associated protein 3 (KIFAP3); unc-13 homolog A (UNC13A); apoptosis-associated tyrosine kinase (AATK); brain-specific angiogenesis inhibitor 1-associated protein 2 (BAIAP2); charged multivesicular body protein 6 (CHMP6); insulin-like growth factor binding protein acid labile subunit (IGFALS); calcium channel, voltage-dependent, T type, alpha 1H subunit (CACNA1H); mitogen-activated protein kinase 8 interacting protein 3 (MAPK8IP3); BAI1-associated protein 3 (BAIAP3); ubiquitin-conjugating enzyme E2I (UBE2I); splA/ryanodine receptor domain and SOCS box containing 3 (SPSB3); Kv channel interacting protein 4 (KCNIP4); potassium channel, voltage gated Shab related subfamily B; member 2 (KCNB2); potassium channel, voltage gated KQT-like subfamily Q, member 5 (KCNQ5); gasdermin D (GSDMD); glutamate receptor, ionotropic, kainate 1 (GRIK1); glutamate receptor, ionotropic, kainate 2 (GRIK2); eukaryotic translation elongation factor 1 delta (EEF1D); ataxin 1 (ATXN1); ataxin 3-like protein (ATXN3L); solute carrier family 1 member 7 (SLC1A7); triadin **(**TRDN); complexin-1 (CPLX1); ankyrin repeat domain 11 (ANKRD11); protein phosphatase 1 regulatory subunit 13B (PPP1R13B); forkhead box O3 (FOXO3); hemochromatosis (HFE); gamma-glutamyltransferase light chain 2 (GGTLC2); autophagy related 7 (ATG7); annexin A5 (ANXA5); gem-associated protein 6 (GEMIN6); myotubularin related protein 7 (MTMR7); acylphosphatase 2 (ACYP2); ZFP14 Zinc Finger Protein (ZFP14); formin 2 (FMN2); ribosomal protein S3A (RPS3A); DEAH-box helicase 30 (DHX30); UDP glycosyltransferase 8 (UGT8); cell adhesion molecule L1 like (CHL1); hyperpolarization-activated cyclic nucleotide-gated channel 1 (HCN1); POTE ankyrin domain family member A (POTEA); olfactory receptor family 4 subfamily A member 5 (OR4A5); olfactory receptor family 4 subfamily C member 12 (OR4C12); NADH dehydrogenase subunit 2 (ND2); cytochrome oxidase subunit-3 (CO3); NADH dehydrogenase subunit 4 (ND4).

## Electronic supplementary material

ESM 1 (GIF 178 kb).

High-resolution image (TIFF 453 kb).

ESM 2 (PDF 560 kb).
