# Supplementary figures and images for: Copy Number Variations in Amyotrophic Lateral Sclerosis: Piecing the Mosaic Tiles Together through a Systems Biology Approach
Source: Mol Neurobiol. 2017 Jan 24;55(2):1299–322. doi: 10.1007/s12035-017-0393-x (PMC5820374; doi:10.1007/s12035-017-0393-x)

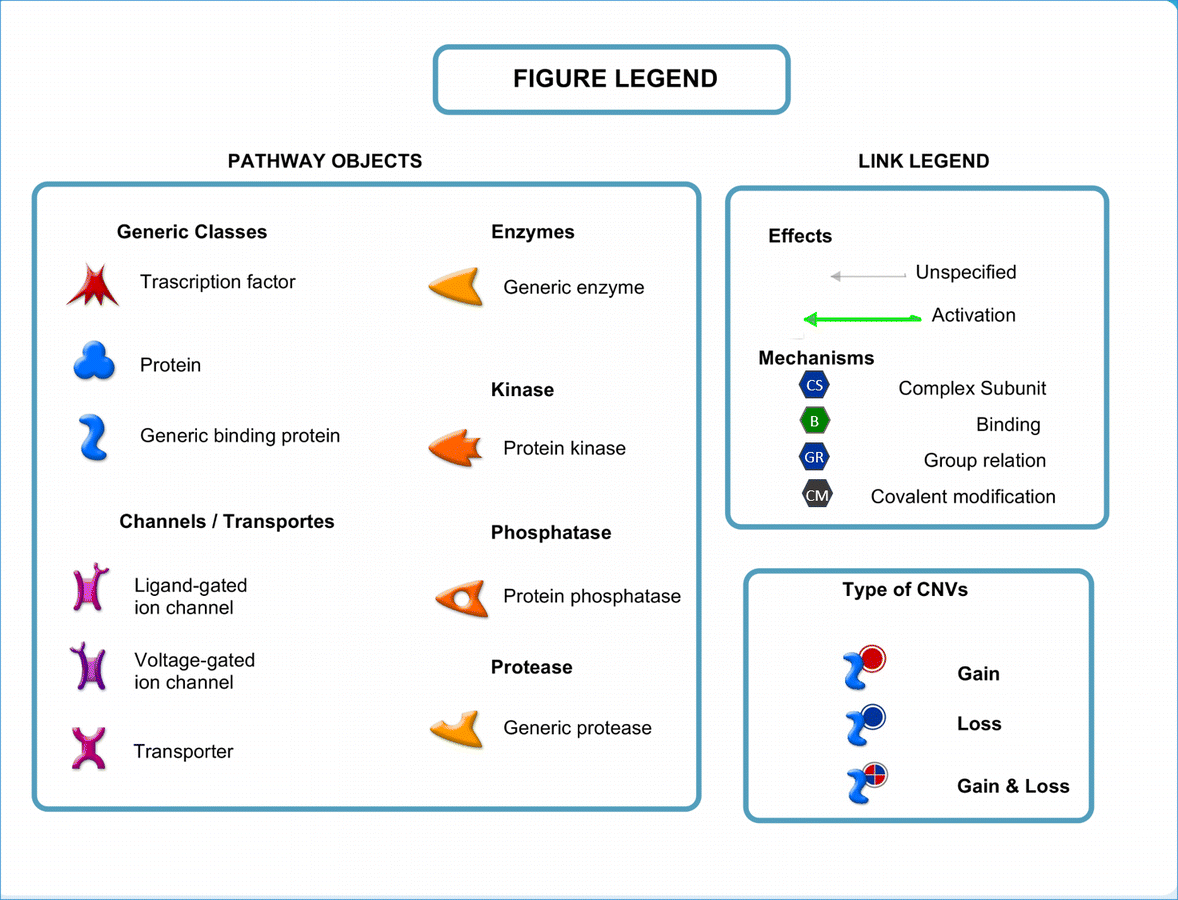

Supplement: Supplementary file 1 — (GIF 178 kb) [file 12035_2017_393_Fig1_ESM.gif]

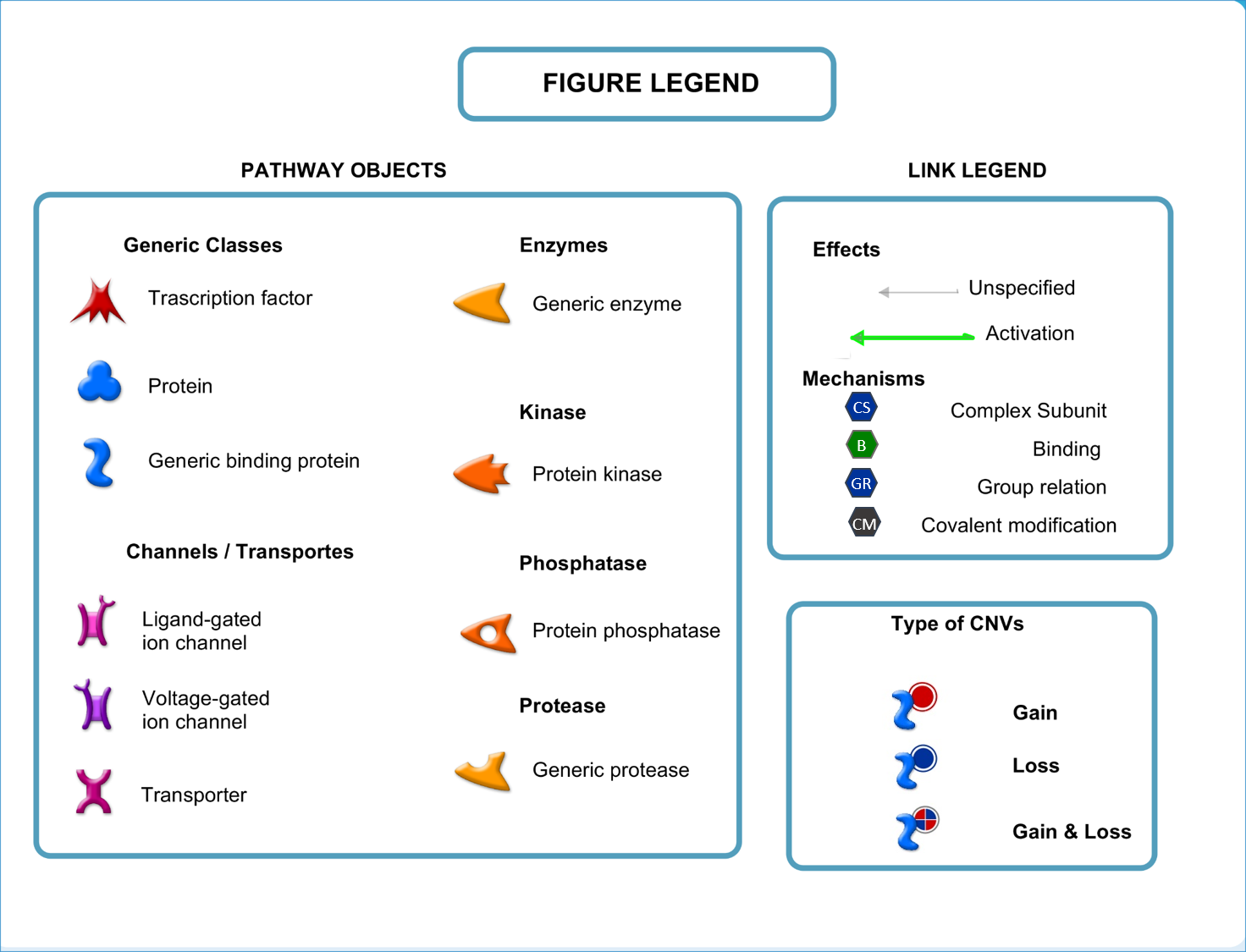

Supplement: Supplementary file 2 — High-resolution image (TIFF 453 kb) [file 12035_2017_393_MOESM1_ESM.tif]
