# Supplementary material for: Copy Number Variations in Amyotrophic Lateral Sclerosis: Piecing the Mosaic Tiles Together through a Systems Biology Approach
Source: Mol Neurobiol. 2017 Jan 24;55(2):1299–322. doi: 10.1007/s12035-017-0393-x (PMC5820374; doi:10.1007/s12035-017-0393-x)
Supplement: Supplementary file 3 — (PDF 560 kb) [file 12035_2017_393_MOESM2_ESM.pdf]

Supplementary Table 1. Genes found to have rare ALS-specific CNVs, not observed in controls individuals of each study nor among >2500 controls reported in the DGV. Genes which may be reasonable ALS candidates are in bold.

Gene	GO biological process	TOT CNVs	Hom. deletions	Het. deletions	Duplications	Reference
**GSDM**	Apoptotic process	15	-	8	7	[Cronin,Blauw et al. 2008; Wain, Pedroso et al. 2009]
LMNTD2	Positive regulation of mRNA splicing, via spliceosome	14	-	8	6	[Cronin,Blauw et al. 2008]
LRRC56	-	14	-	8	6	[Cronin,Blauw et al. 2008]
RASSF7	Apoptotic process, signal transduction	13	-	7	6	[Cronin,Blauw et al. 2008]
CDHR5	Cell adhesion	12	-	6	6	[Cronin,Blauw et al. 2008]
DRD4	G-protein coupled receptor signaling pathway, positive regulation of excitatory postsynaptic potential, regulation of calcium-mediated signaling, regulation of neurotransmitter secretion, short-term memory, signal transduction, synaptic transmission	12	-	6	6	[Cronin,Blauw et al. 2008]
IRF7	Cytokine-mediated signaling pathway, regulation of immune response, transcription	12	-	6	6	[Cronin,Blauw et al. 2008]
PHRF1	Intracellular signal transduction, mRNA processing, transcription	12	-	6	6	[Cronin,Blauw et al. 2008]
SCT	Brain development, negative regulation of neuron apoptotic process	12	-	6	6	[Cronin,Blauw et al. 2008]
**EEF1D**	Cellular protein metabolic process, gene expression, mRNA transcription, positive regulation of I-kappaB kinase/NF-kappaB signaling, regulation of cell death, signal transduction	10	-	7	3	[Cronin,Blauw et al. 2008]
MROH6	-	9	-	6	3	[Cronin,Blauw et al. 2008]
NAPRT1	Pyridine nucleotide biosynthetic process, response to oxidative stress	9	-	6	3	[Cronin,Blauw et al. 2008]
C14orf2	-	7	-	7	-	[Cronin,Blauw et al. 2008]
DEAF1	Nervous system development, transcription	7	-	6	1	[Cronin,Blauw et al. 2008]
**PPP1R13B**	Apoptotic process	7	-	7	-	[Cronin,Blauw et al. 2008]
C14orf177	-	6	-	6	-	[Wain, Pedroso et al. 2009]
**ACYP2**	Phosphate-containing compound metabolic process	5	-	5	-	[Uyan, Omur et al. 2013; Blauw, Barnes et al. 2008]
**ADGRL3**	G-protein coupled receptor signaling pathway, brain development, cell-cell adhesion, neuron migration, signal transduction, synapse assembly	4	-	-	4	[Uyan, Omur et al. 2013]
BTN1A1	Negative regulation of activated T cell proliferation, negative regulation of cellular metabolic process, negative regulation of cytokine secretion	4	-	4	-	[Wain, Pedroso et al. 2009;Blauw, Barnes et al. 2008]
**CSMD1**	Glucose homeostasis	4	4	-	-	[Wain, Pedroso et al. 2009; [Pamphlett, Morahan et al. 2011]]
OR3A1	G-protein coupled receptor signaling pathway, response to stimulus, signal transduction	4	-	4	-	[Wain, Pedroso et al. 2009;Blauw, Barnes et al. 2008]
OR3A2	G-protein coupled receptor signaling pathway, response to stimulus, signal transduction	4	-	4	-	[Wain, Pedroso et al. 2009;Blauw, Barnes et al. 2008]
OR3A4	G-protein coupled receptor signaling pathway, response to stimulus, signal transduction	4	-	4	-	[Wain, Pedroso et al. 2009;Blauw, Barnes et al. 2008]
ADK	Adenosine metabolic process, phosphorylation, positive regulation of T cell proliferation,small molecule metabolic process	3	-	3	-	[Wain, Pedroso et al. 2009; Blauw, Barnes et al. 2008]

PUDP	Dephosphorylation, metabolic process	3	-	1	2	[Wain, Pedroso et al. 2009]
SLCO1B1	Ion transport, metabolic process, transmembrane transport	3	-	2	1	[Wain, Pedroso et al. 2009]
STS	Learning or memory, metabolic process, response to pH, steroid metabolic process	3	-	1	2	[Wain, Pedroso et al. 2009]
ZNF326	RNA splicing, regulation of transcription	3	-	-	3	[Blauw, Al-Chalabi et al. 2010; Wain, Pedroso et al. 2009]
ARL8B	Cell cycle, cell division	2	-	2	-	[Blauw, Al-Chalabi et al. 2010; Blauw, Barnes et al. 2008]
**ATXN1**	RNA processing, excitatory postsynaptic potential, regulation of transcription, regulation of glial cell proliferation	2	-	2	-	[Wain, Pedroso et al. 2009]
BTN2A1	Lipid metabolic process	2	-	2	-	[Wain, Pedroso et al. 2009]
C9orf114	Cell cycle, cell division, methylation	2	-	2	-	[Wain, Pedroso et al. 2009]
CALHM2	Ion transmembrane transport	2	-	1	1	[Blauw, Al-Chalabi et al. 2010]
CALHM3	Ion transmembrane transport	2	-	1	1	[Blauw, Al-Chalabi et al. 2010]
CCBL1	Biosynthetic process	2	-	2	-	[Wain, Pedroso et al. 2009]
CDH7	Cell adhesion	2	-	2	-	[Blauw, Al-Chalabi et al. 2010; Blauw, Barnes et al. 2008]
CFAP52	Signal transduction, RNA processing, remodeling the cytoskeleton, regulation of vesicular traffic, cell division	2	-	2	-	[Blauw, Al-Chalabi et al. 2010; Blauw, Barnes et al. 2008]
CHCHD6	Cellular response to DNA damage stimulus	2	-	2	-	[Blauw, Barnes et al. 2008]
**CHMP6**	Endosomal transport, membrane organization, nucleus organization, protein transport	2	-	-	2	[Blauw, Al-Chalabi et al. 2010; Pamphlett, Morahan et al. 2011]
CLRN1	Actin filament organization, cell motility, neuromuscular process controlling balance, response to stimulus	2	-	-	2	[Blauw, Al-Chalabi et al. 2010]
**COX7C**	Cellular metabolic process, gene expression, ion transmembrane transport, respiratory electron transport chain	2	-	2	-	[Blauw, Al-Chalabi et al. 2010; Blauw, Barnes et al. 2008]
DBH	Catecholamine metabolic process, memory,oxidation-reduction process, regulation of cell proliferation and apoptotic signaling pathway, response to pain, metabolic process, synaptic transmission	2	-	2	-	[Wain, Pedroso et al. 2009]
DERA	Catabolic process, small molecule metabolic process	2	-	2	-	[Blauw, Al-Chalabi et al. 2010] [Blauw, Barnes et al. 2008]
EDEM1	Cellular protein metabolic process, endoplasmic reticulum unfolded protein response, protein folding	2	-	2	-	[Blauw, Al-Chalabi et al. 2010; Blauw, Barnes et al. 2008]
EGFL6	Cell adhesion, cell cycle, cell differentiation, extracellular matrix organization	2	-	-	2	[Wain, Pedroso et al. 2009]
ENDOG	DNA catabolic process, cellular response to calcium ion, cellular response to glucose stimulus, cellular response to hypoxia, cellular response to oxidative stress, regulation of apoptotic process	2	-	2	-	[Wain, Pedroso et al. 2009]
EPS8	Actin cytoskeleton reorganization, cell proliferation, signal transduction	2	-	2	-	[Blauw, Al-Chalabi et al. 2010; Blauw, Barnes et al. 2008]
FAM135B	Cellular lipid metabolic process	2	-	2	-	[Wain, Pedroso et al. 2009]
FAM49B	-	2	-	-	2	[Blauw, Al-Chalabi et al. 2010]
**HFE**	Antigen processing and presentation, immune response, ion transport, regulation of proteasomal ubiquitin-dependent protein catabolic process, regulation of receptor activity, positive regulation of gene expression, regulation of protein binding, transport	2	-	-	2	[Wain, Pedroso et al. 2009]
HINT3	Metabolic process	2	-	2	-	[Blauw, Al-Chalabi et al. 2010; Blauw, Barnes et al. 2008]
JRKL	Central nervous system development	2	-	1	1	[Blauw, Al-Chalabi et al. 2010; Blauw, Barnes et al. 2008]

LAMA1	Axon guidance, cell adhesion, neuron projection development, protein phosphorylation, regulation of cell adhesion, regulation of cell migration, tissue development	2	-	1	1	[Wain, Pedroso et al. 2009]
**MTMR7**	Dephosphorylation	2	-	2	-	[Blauw, Al-Chalabi et al. 2010; Blauw, Barnes et al. 2008]
NEK11	Cell cycle, intracellular signal transduction, protein phosphorylation	2	-	2	-	[Blauw, Barnes et al. 2008]
OR4M2	G-protein coupled receptor signaling pathway, response to stimulus, signal transduction	2	-	2	-	[Pamphlett, Morahan et al. 2011]
OR4N4	G-protein coupled receptor signaling pathway, response to stimulus,signal transduction	2	-	2	-	[Pamphlett, Morahan et al. 2011]
OR6P1	Neuronal response to olfactory stimulus	2	-	-	2	[Wain, Pedroso et al. 2009]
OR6Y1	Neuronal response to olfactory stimulus	2	-	-	2	[Wain, Pedroso et al. 2009]
PART1_HUMAN	-	2	-	2	-	[Blauw, Barnes et al. 2008]
PDCD11	RNA processing	2	-	1	1	[Blauw, Al-Chalabi et al. 2010]
RALGAPA1	Activation of GTPase activity, signal transduction, regulation of transcription	2	-	2	-	[Wain, Pedroso et al. 2009]
RGPD6	Intracellular transport, mRNA transport, protein folding	2	-	2	-	[Blauw, Barnes et al. 2008]
RIMS2	cAMP-mediated signaling, calcium ion regulated exocytosis, cell differentiation, exocytosis, intracellular protein transport, neurotransmitter secretion and transport, positive regulation of gene expression, regulation of membrane potential, transport	2	-	2	-	[Wain, Pedroso et al. 2009]
SERPINB12	Hematopoietic progenitor cell differentiation, regulation of protein catabolic process	2	-	2	-	[Blauw, Al-Chalabi et al. 2010; Blauw, Barnes et al. 2008]
SERPINB13	Hematopoietic progenitor cell differentiation, regulation of protein catabolic process	2	-	2	-	[Blauw, Al-Chalabi et al. 2010; Blauw, Barnes et al. 2008]
SLC25A43	Transmembrane transport	2	-	2	-	[Wain, Pedroso et al. 2009]
SOAT2	Cholesterol homeostasis	2	-	2	-	[Blauw, Barnes et al. 2008]
SRFBP1	Regulation of transcription	2	-	2	-	[Blauw, Al-Chalabi et al. 2010; Blauw, Barnes et al. 2008]
SYK	Cell differentiation, cell proliferation, intracellular signal transduction, cell-cell adhesion, regulation of calcium-mediated signaling, regulation of cell adhesion, regulation of immune response	2	-	1	1	[Wain, Pedroso et al. 2009]
TAF5	Transcription	2	-	1	1	[Blauw, Al-Chalabi et al. 2010]
TBC1D13	Regulation of GTPase activity	2	-	2	-	[Wain, Pedroso et al. 2009]
TEX101	Regulation of leukocyte activation, regulation of release of sequestered calcium ion into cytosol	2	-	2	-	[Wain, Pedroso et al. 2009]
TRIM32	Actin ubiquitination, axon development, innate immune response, muscle cell cellular homeostasis, regulation of intrinsic apoptotic signaling pathway in response to DNA damage, regulation of cell growth and migration, regulation of neuron differentiation, protein catabolic process	2	-	2	-	[Blauw, Al-Chalabi et al. 2010]
UCP1	Cellular metabolic process, mitochondrial transport, regulation of transcription	2	-	2	-	[Wain, Pedroso et al. 2009]
USMG5	-	2	-	1	1	[Blauw, Al-Chalabi et al. 2010]
YWHAE	Apoptotic process, gene expression, signal transduction, neuron migration, signal transduction, transcription	2	-	1	1	[Blauw, Al-Chalabi et al. 2010]
ZNF578	Regulation of transcription	2	-	2	-	[Blauw, Barnes et al. 2008]

ZNF808	Regulation of transcription	2	-	2	-	[Blauw, Barnes et al. 2008]
ABHD4	Lipid metabolic process	1	-	1	-	[Blauw, Al-Chalabi et al. 2010]
ACSM6	Lipid metabolic process, regulation of protein targeting to mitochondrion, regulation of protein stability	1	-	-	1	[Blauw, Al-Chalabi et al. 2010]
ACTN3	Focal adhesion assembly, muscle contraction	1	-	1	-	[Blauw, Barnes et al. 2008]
AHNAK2	Plasma membrane repair	1	-	-	1	[Blauw, Al-Chalabi et al. 2010]
AK8	Phosphorylation, small molecule metabolic process,	1	-	1	-	[Blauw, Barnes et al. 2008]
ALOX15	Apoptosis, cellular response to calcium ion, lipid metabolic process, regulation of immune response, oxidation-reduction process, regulation of actin filament polymerization, regulation of cell growth, positive regulation of cell proliferation, regulation of membrane potential, response to endoplasmic reticulum stress	1	-	1	-	[Blauw, Al-Chalabi et al. 2010]
ANKRD36B	-	1	1	-	-	[Pamphlett, Morahan et al. 2011]
ANKRD37	Protein binding	1	-	-	1	[Blauw, Al-Chalabi et al. 2010]
**ANXA5**	Calcium ion transmembrane transport, regulation of apoptotic process, signal transduction	1	-	1	-	[Blauw, Barnes et al. 2008]
APCDD1	Wnt signaling pathway, astrocyte cell migration	1	-	-	1	[Blauw, Al-Chalabi et al. 2010]
ARHGAP18	Regulation of GTPase activity, regulation of actin cytoskeleton organization and polymerization, regulation of cell motility, signal transduction	1	-	-	1	[Blauw, Al-Chalabi et al. 2010]
ARHGEF19	Wnt signaling pathway, regulation of actin cytoskeleton organization	1	-	-	1	[Blauw, Al-Chalabi et al. 2010]
ARHGEF4	Apoptotic signaling pathway, intracellular signal transduction	1	-	-	1	[Blauw, Al-Chalabi et al. 2010]
ARNTL	Regulation of gene expression, regulation of transcription, regulation of Wnt signaling pathway, regulation of skeletal muscle cell differentiation, proteasome-mediated ubiquitin-dependent protein catabolic process, regulation of cell cycle, regulation of neurogenesis, response to redox state	1	1	-	-	[Pamphlett, Morahan et al. 2011]
ARPP19	Cell cycle, cell division, signal transduction	1	-	1	-	[Blauw, Al-Chalabi et al. 2010]
ARRB2	Notch signaling pathway, brain development, cell chemotaxis, endocytosis, regulation of release of cytochrome c from mitochondria, regulation of apoptotic process, regulation of protein ubiquitination, regulation of synaptic transmission, regulation of protein phosphorylation, signal transduction, transcription, transport	1	-	1	-	[Blauw, Al-Chalabi et al. 2010]
ARRDC4	Positive regulation of ubiquitin-protein transferase activity	1	-	-	1	[Blauw, Al-Chalabi et al. 2010]
ARSJ	Cellular protein metabolic process	1	-	1	-	[Blauw, Al-Chalabi et al. 2010]
**ATG7**	Autophagy, cellular homeostasis, cellular protein modification process, cellular response to hyperoxia, central nervous system neuron axonogenesis, cerebral cortex development, membrane organization, mitochondrion organization, regulation of apoptotic process, protein transport, protein ubiquitination	1	-	1	-	[Pamphlett, Morahan et al. 2011]
ATRN	Cerebellum development, inflammatory response, response to oxidative stress	1	-	-	1	[Blauw, Al-Chalabi et al. 2010]
**ATXN3L**	Cellular response to misfolded protein, protein deubiquitination, regulation of transcription	1	-	-	1	[Wain, Pedroso et al. 2009]

AXIN2	Wnt signaling pathway, cell death, cell differentiation, cell proliferation, mRNA stabilization, regulation of GTPase activity, regulation of transcription	1	-	1	-	[Blauw, Al-Chalabi et al. 2010]
BARHL1	Midbrain development, regulation of neuron apoptotic process, neuron migration, regulation of transcription	1	-	-	1	[Blauw, Al-Chalabi et al. 2010]
BBS1	Protein transport, cellular lipid metabolic process, cerebral cortex development, microtubule cytoskeleton organization, neuron migration, protein localization, response to stimulus, transport	1	-	1	-	[Blauw, Barnes et al. 2008]
BBS7	Protein transport, cellular lipid metabolic process, cerebral cortex development, microtubule cytoskeleton organization, neuron migration, protein localization, response to stimulus, transport	1	-	1	-	[Blauw, Barnes et al. 2008]
BPIFA1	Immune system process, regulation of ion transmembrane transport	1	-	1	-	[Blauw, Barnes et al. 2008]
BPIFA3	-	1	-	1	-	[Blauw, Barnes et al. 2008]
BPIFA4P	-	1	-	1	-	[Blauw, Barnes et al. 2008]
BRCA2	DNA damage response, signal transduction, brain development, cell aging, cell cycle, cell proliferation, cellular response to DNA damage stimulus, apoptotic signaling pathway, regulation of cell proliferation, regulation of cytokinesis, regulation of transcription	1	-	1	-	[Blauw, Al-Chalabi et al. 2010]
BSPRY	Ion transport	1	-	-	1	[Blauw, Al-Chalabi et al. 2010]
C14orf79	-	1	-	-	1	[Blauw, Al-Chalabi et al. 2010]
C21orf58	-	1	-	-	1	[Blauw, Al-Chalabi et al. 2010]
C3orf22	-	1	-	-	1	[Blauw, Al-Chalabi et al. 2010]
C4orf3	-	1	-	1	-	[Blauw, Al-Chalabi et al. 2010]
C4orf47	-	1	-	-	1	[Blauw, Al-Chalabi et al. 2010]
C5orf38	-	1	-	-	1	[Blauw, Al-Chalabi et al. 2010]
C9orf79	Cell differentiation	1	-	-	1	[Blauw, Al-Chalabi et al. 2010]
C9orf9	-	1	-	-	1	[Blauw, Al-Chalabi et al. 2010]
CACNA2D1	Calcium ion transmembrane transport	1	-	1	-	[Blauw, Barnes et al. 2008]
CCDC110	-	1	-	-	1	[Blauw, Al-Chalabi et al. 2010]
CCDC64	Nervous system development, transport	1	1	-	-	[Pamphlett, Morahan et al. 2011]
CCDC82	-	1	-	1	-	[Blauw, Barnes et al. 2008]
CCL3L3	Chemotaxis, astrocyte cell migration, calcium ion transport, calcium-mediated signaling, cell activation, cell-cell signaling, cellular calcium ion homeostasis, chemotaxis, cytoskeleton organization, exocytosis, immune response, inflammatory response, regulation of cell proliferation, regulation of gene expression, regulation of cell migration	1	-	-	1	[Pamphlett, Morahan et al. 2011]
CCL4L2 (+promoter)	Signaling pathway, chemotaxis, immune response, inflammatory response	1	-	-	1	[Pamphlett, Morahan et al. 2011]
CCNA2	Cell cycle, cell division, cellular response to hypoxia, regulation of transcription	1	-	1	-	[Blauw, Barnes et al. 2008]

CCND1	Mitotic cell cycle, Notch signaling pathway, Wnt signaling pathway, cell cycle, cell division, cellular response to DNA damage stimulus, regulation of transcription, response to calcium ion	1	-	-	1	[Blauw, Al-Chalabi et al. 2010]
CCR4	Chemotaxis, immune response, inflammatory response, neuron migration, regulation of ion concentration, signal transduction	1	-	-	1	[Blauw, Al-Chalabi et al. 2010]
CD83	Defense response, signal transduction	1	-	1	-	[Blauw, Al-Chalabi et al. 2010]
CDCA2	Cell cycle, cell division, regulation of protein dephosphorylation	1	-	1	-	[Blauw, Al-Chalabi et al. 2010]
CDK20	Cell cycle	1	-	-	1	[Blauw, Al-Chalabi et al. 2010]
CDK3	Cell cycle, cell division, cell proliferation, cellular response to DNA damage stimulus, protein phosphorylation	1	-	1	-	[Blauw, Barnes et al. 2008]
**CDR1**	-	1	-	-	1	[Schoichet, Waibel et al. 2009]
CDV3	Cell proliferation	1	-	-	1	[Blauw, Al-Chalabi et al. 2010]
CEMIP	Cell migration, regulation of protein kinase C activity, positive regulation of release of sequestered calcium ion into cytosol, small molecule metabolic process	1	-	-	1	[Pamphlett, Morahan et al. 2011]
CHAC2	Glutathione biosynthetic process, small molecule metabolic process	1	-	1	-	[Blauw, Al-Chalabi et al. 2010]
CHST13	Metabolic process	1	-	-	1	[Blauw, Al-Chalabi et al. 2010]
CIT	Golgi organization, cell cycle, cell differentiation, cell division, cytokinesis, dendrite development, generation of neurons, intracellular signal transduction, regulation of neuron differentiation, nervous system development	1	-	-	1	[Blauw, Al-Chalabi et al. 2010]
CLIP2	Negative regulation of microtubule depolymerization	1	-	1	-	[Blauw, Al-Chalabi et al. 2010]
CMTM7	Cell differentiation, chemotaxis	1	-	1	-	[Blauw, Al-Chalabi et al. 2010]
COG3	ER to Golgi vesicle-mediated transport, Golgi organization, cellular protein metabolic process, membrane organization, post-translational protein modification	1	-	-	1	[Blauw, Al-Chalabi et al. 2010]
COL7A1	ER to Golgi vesicle-mediated transport, cell adhesion, cellular protein metabolic process, extracellular matrix organization, membrane organization, post-translational protein modification	1	-	1	-	[Blauw, Barnes et al. 2008]
COLEC12	Immune response, regulation of cell adhesion	1	-	-	1	[Blauw, Al-Chalabi et al. 2010]
COLGALT2	Extracellular matrix organization, metabolic process	1	-	-	1	[Blauw, Al-Chalabi et al. 2010]
CRISPLD1	Face morphogenesis	1	-	1	-	[Blauw, Al-Chalabi et al. 2010]
CRTAM	Rgulation of immune response, cell adhesion	1	-	-	1	[Blauw, Al-Chalabi et al. 2010]
CSAD	Metabolic process	1	-	1	-	[Blauw, Barnes et al. 2008]
CSTF2T	mRNA processing	1	-	-	1	[Blauw, Al-Chalabi et al. 2010]
CTBS	Metabolic process	1	-	-	1	[Blauw, Al-Chalabi et al. 2010]
CXCL16	Cell chemotaxis, regulation of cell growth, regulation of cell migration	1	-	1	-	[Blauw, Al-Chalabi et al. 2010]
CYP2C8	Oxidative demethylation, metabolic process	1	-	-	1	[Blauw, Al-Chalabi et al. 2010]
CYP4X1	Oxidation-reduction process	1	-	-	1	[Blauw, Al-Chalabi et al. 2010]
DAD1	Apoptotic process, cellular protein metabolic process, protein glycosylation	1	-	1	-	[Blauw, Al-Chalabi et al. 2010]
DAGLB	Lipid metabolic process,neurogenesis, neurotransmitter biosynthetic process, transport	1	-	-	1	[Blauw, Al-Chalabi et al. 2010]

DCBLD2	Intracellular receptor signaling pathway, regulation of cell growth	1	1	-	-	[Blauw, Al-Chalabi et al. 2010]
DDX31	Ribosome biogenesis	1	-	-	1	[Blauw, Al-Chalabi et al. 2010]
DDX54	RNA processing, signaling pathway, transcription	1	-	1	-	[Blauw, Al-Chalabi et al. 2010]
DEFB123	Immune response	1	-	-	1	[Blauw, Al-Chalabi et al. 2010]
DEFB124	Immune response	1	-	-	1	[Blauw, Al-Chalabi et al. 2010]
DEPDC1B	Cell migration,signal transduction	1	-	1	-	[Blauw, Barnes et al. 2008]
DGKI	Intracellular signal transduction, regulation of GTPase activity, neurotransmitter secretion, phosphorylation, regulation of synaptic transmission	1	1	-	-	[Pamphlett, Morahan et al. 2011]
DHX35	RNA processing	1	-	1	-	[Blauw, Al-Chalabi et al. 2010]
DHX57	RNA processing	1	-	1	-	[Blauw, Barnes et al. 2008]
DMRT2	Regulation of transcription	1	-	-	1	[Blauw, Al-Chalabi et al. 2010]
DMRT3	Regulation of transcription, cell differentiation, transmission of nerve impulse	1	-	-	1	[Blauw, Al-Chalabi et al. 2010]
DNAH8	Metabolic process, microtubule-based movement	1	-	1	-	[Blauw, Al-Chalabi et al. 2010]
DPP8	Immune response, proteolysis	1	-	1	-	[Blauw, Al-Chalabi et al. 2010]
DRG2	Signal transduction	1	-	1	-	[Blauw, Al-Chalabi et al. 2010]
DSEL	Metabolic process	1	-	1	-	[Blauw, Barnes et al. 2008]
DYDC1	Chromatin silencing at telomere, histone H3-K4 methylation	1	-	-	1	[Blauw, Al-Chalabi et al. 2010]
DYDC2	Chromatin silencing at telomere, histone H3-K4 methylation	1	-	-	1	[Blauw, Al-Chalabi et al. 2010]
ECHDC3	Metabolic process	1	-	-	1	[Blauw, Al-Chalabi et al. 2010]
EDA2R	Cell differentiation, intrinsic apoptotic signaling pathway	1	-	1	-	[Blauw, Barnes et al. 2008]
EDNRA	G-protein coupled receptor signaling pathway, signal transduction, aging, cell proliferation, cellular response to stimulus, endothelin receptor signaling pathway, regulation of apoptotic process, regulation of calcium ion transport, regulation of inflammatory response, response to hypoxia	1	-	-	1	[Blauw, Al-Chalabi et al. 2010]
ELMOD2	Defense response to virus, positive regulation of GTPase activity	1	-	1	-	[Wain, Pedroso et al. 2009]
ELOVL7	Cellular lipid metabolic process	1	-	1	-	[Blauw, Barnes et al. 2008]
EPHA2	Angiogenesis, apoptotic process, axon guidance, cell adhesion, cell chemotaxis, cell differentiation, cell migration, defense response, inflammatory response, neuron differentiation, response to growth factor, skeletal system development	1	-	-	1	[Blauw, Al-Chalabi et al. 2010]
ERCC8	DNA repair, protein ubiquitination, response to oxidative stress	1	-	1	-	[Blauw, Barnes et al. 2008]
ETS1	Cell differentiation, cell motility, immune system process, regulation of cell cycle, regulation of cell proliferation, regulation of inflammatory response, regulation of angiogenesis, positive regulation of cell migration, regulation of apoptotic process, regulation of extracellular matrix, response to hypoxia, transcription	1	-	1	-	[Pamphlett, Morahan et al. 2011]
EVPL	Epidermis development	1	-	1	-	[Blauw, Barnes et al. 2008]
EXO5	DNA repair, cellular response to DNA damage stimulus	1	-	1	-	[Blauw, Barnes et al. 2008]

EXOC4	Golgi to transport vesicle transport, cellular protein metabolic process, membrane biogenesis, membrane organization, oligodendrocyte differentiation, organelle organization, regulation of calcium-mediated signaling, protein transport, synaptic transmission	1	-	-	1	[Blauw, Al-Chalabi et al. 2010]
EXOSC9	RNA processing	1	-	1	-	[Blauw, Barnes et al. 2008]
FABP2	Fatty acid metabolic process and transport	1	-	1	-	[Blauw, Al-Chalabi et al. 2010]
FAM114A1	-	1	-	1	-	[Blauw, Al-Chalabi et al. 2010]
FAM150A	-	1	-	-	1	[Blauw, Al-Chalabi et al. 2010]
FAM169A	-	1	-	1	-	[Blauw, Al-Chalabi et al. 2010]
FAM213A	Cellular oxidation-reduction process	1	-	-	1	[Blauw, Al-Chalabi et al. 2010]
FAM214A	-	1	-	1	-	[Blauw, Al-Chalabi et al. 2010]
FAM26D	Ion transmembrane transport	1	-	1	-	[Blauw, Al-Chalabi et al. 2010]
FAM83D	Cell cycle, cell division, cell migration, cell proliferation	1	-	1	-	[Blauw, Al-Chalabi et al. 2010]
FAM92B	-	1	-	-	1	[Blauw, Al-Chalabi et al. 2010]
FASTKD5	Cellular respiration, mRNA processing, mitochondrial RNA processing, protein phosphorylation	1	-	1	-	[Blauw, Al-Chalabi et al. 2010]
FBXO42	Protein ubiquitin-proteasome system	1	-	-	1	[Blauw, Al-Chalabi et al. 2010]
FMNL2	Actin cytoskeleton organization, signal transduction	1	-	1	-	[Blauw, Barnes et al. 2008]
FNDC3B	Cell migration and differentiation	1	-	-	1	[Blauw, Al-Chalabi et al. 2010]
FOXS1	Cell differentiation, neuromuscular process controlling balance, regulation of transcription	1	-	-	1	[Blauw, Al-Chalabi et al. 2010]
FUT3	Cell-cell recognition, cellular protein metabolic process, post-translational protein modification	1	-	-	1	[Blauw, Al-Chalabi et al. 2010]
FUT5	Protein glycosylation	1	-	-	1	[Blauw, Al-Chalabi et al. 2010]
FUT6	Protein glycosylation	1	-	-	1	[Blauw, Al-Chalabi et al. 2010]
GATA4	Signal transduction, transcription, Wnt signaling pathway, cardiac muscle cell differentiation, cell development, cell-cell signaling, regulation of angiogenesis, regulation of gene expression, skeletal muscle cell differentiation,	1	-	-	1	[Blauw, Al-Chalabi et al. 2010]
**GEMIN6**	Gene expression, mRNA processing, mRNA splicing	1	-	1	-	[Blauw, Barnes et al. 2008]
GFI1B	Cell proliferation, chromatin modification, regulation of transcription	1	-	-	1	[Blauw, Al-Chalabi et al. 2010]
GFRA4	Glial cell-derived neurotrophic factor receptor signaling pathway, transmembrane receptor protein tyrosine kinase signaling pathway	1	-	-	1	[Blauw, Al-Chalabi et al. 2010]
GINS4	DNA replication	1	-	-	1	[Blauw, Al-Chalabi et al. 2010]
GJB6	Apoptotic process, cell communication, regulation of cell proliferation, response to electrical stimulus	1	-	1	-	[Blauw, Barnes et al. 2008]
GLP1R	Learning or memory, regulation of neuron apoptotic process, neuropeptide signaling pathway, regulation of cell differentiation, regulation of cell proliferation, regulation of transcription, regulation of calcium ion transport, response to stress, signal transduction, metabolic process	1	-	1	-	[Blauw, Al-Chalabi et al. 2010]
GNAQ	Regulation of apoptotic process, neuron remodeling, signaling pathway, positive regulation of apoptotic process, protein stabilization, signal transduction, skeletal system development	1	-	1	-	[Blauw, Al-Chalabi et al. 2010]

GOLGA4	Regulation of axon extension, vesicle-mediated transport	1	-	1	-	[Blauw, Al-Chalabi et al. 2010]
GOLGA7	Golgi to plasma membrane protein transport	1	-	-	1	[Blauw, Al-Chalabi et al. 2010]
GOLT1B	Protein transport, signal transduction	1	-	1	-	[Blauw, Al-Chalabi et al. 2010]
GPR37	G-protein coupled receptor signaling pathway, dopamine biosynthetic process, regulation of cell death, signal transduction	1	-	1	-	[Blauw, Al-Chalabi et al. 2010]
**GRIK1**	Central nervous system development, glutamate receptor signaling pathway, ion transport, membrane depolarization, regulation of synaptic transmission, regulation of synaptic plasticity	1	-	1	-	[Pamphlett, Morahan et al. 2011]
**GRIK2**	Central nervous system development, glutamate receptor signaling pathway, ion transport, membrane depolarization, regulation of synaptic transmission, regulation of synaptic plasticity, regulation of neuron apoptotic process, regulation of JNK cascade	1	-	-	1	[Pamphlett, Morahan et al. 2011]
GTF2F2	RNA splicing, gene expression, regulation of transcription	1	-	-	1	[Blauw, Al-Chalabi et al. 2010]
GTF3C4	Regulation of catalytic activity, RNA splicing, gene expression, regulation of transcription	1	-	-	1	[Blauw, Al-Chalabi et al. 2010]
GUCY2C	Cyclic nucleotide biosynthetic process, signal transduction, protein phosphorylation, regulation of cell proliferation	1	-	1	-	[Blauw, Al-Chalabi et al. 2010]
H2AFJ	Chromatin silencing	1	-	1	-	[Blauw, Al-Chalabi et al. 2010]
HACD3	JNK cascade, signal transduction, lipid metabolic process	1	-	1	-	[Blauw, Al-Chalabi et al. 2010]
HDHD3	Metabolic process	1	-	-	1	[Blauw, Al-Chalabi et al. 2010]
HIST4H4	DNA replication-dependent nucleosome assembly, nucleosome assembly, protein heterotetramerization	1	-	1	-	[Blauw, Al-Chalabi et al. 2010]
HS3ST1	Metabolic process	1	-	-	1	[Blauw, Al-Chalabi et al. 2010]
HSPA12B	-	1	-	-	1	[Blauw, Al-Chalabi et al. 2010]
HTATIP2	Angiogenesis, apoptotic process, cell differentiation, oxidation-reduction process, regulation of transcription, protein autophosphorylation	1	-	-	1	[Blauw, Al-Chalabi et al. 2010]
HTR1B	Behavior, bone remodeling, regulation of synaptic transmission, signal transduction	1	-	-	1	[Blauw, Al-Chalabi et al. 2010]
IBSP	Cell adhesion, cellular response to growth factor stimulus, extracellular matrix organization, integrin-mediated signaling pathway, ossification	1	-	-	1	[Blauw, Al-Chalabi et al. 2010]
IFNAR1	JAK-STAT cascade,cytokine-mediated signaling pathway, regulation of transcription	1	-	1	-	[Blauw, Al-Chalabi et al. 2010]
IGDCC3	Neuromuscular process controlling balance	1	-	1	-	[Blauw, Al-Chalabi et al. 2010]
IP6K2	Cytokine-mediated signaling pathway, metabolic process, regulation of cell growth, phosphate ion transport, phosphorylation, regulation of apoptotic process	1	-	1	-	[Blauw, Barnes et al. 2008]
IQCD	-	1	-	1	-	[Blauw, Al-Chalabi et al. 2010]
IRX2	Metanephros development, regulation of transcription	1	-	-	1	[Blauw, Al-Chalabi et al. 2010]
ISM2	-	1	-	-	1	[Blauw, Al-Chalabi et al. 2010]
ITGB6	Cell adhesion, extracellular matrix organization, inflammatory response, integrin-mediated signaling pathway	1	-	-	1	[Blauw, Al-Chalabi et al. 2010]

ITGB7	Cell adhesion, integrin-mediated signaling pathway, leukocyte migration	1	-	1	-	[Blauw, Barnes et al. 2008]
**KCNIP4**	Potassium ion transport	1	1	-	-	[Pamphlett, Morahan et al. 2011]
KCNQ3	Axon guidance, ion transmembrane transport, membrane hyperpolarization, oligodendrocyte development, synaptic transmission	1	-	-	1	[Blauw, Al-Chalabi et al. 2010]
**KCNQ5**	Potassium ion transport, synaptic transmission	1	-	1	-	[Pamphlett, Morahan et al. 2011]
KCTD4	Protein homooligomerization	1	-	-	1	[Blauw, Al-Chalabi et al. 2010]
KIAA0513	-	1	-	-	1	[Blauw, Al-Chalabi et al. 2010]
KLB	Axon guidance, signaling pathway, innate immune response	1	-	-	1	[Blauw, Al-Chalabi et al. 2010]
KRTAP20-1	-	1	-	1	-	[Blauw, Al-Chalabi et al. 2010]
KRTAP20-2	-	1	-	1	-	[Blauw, Al-Chalabi et al. 2010]
KRTAP20-3	-	1	-	1	-	[Blauw, Al-Chalabi et al. 2010]
KRTAP6-1	-	1	-	1	-	[Blauw, Al-Chalabi et al. 2010]
L3MBTL3	Chromatin modification, regulation of transcription	1	-	-	1	[Blauw, Al-Chalabi et al. 2010]
LAMC1	Axon guidance, cell adhesion, cell migration, extracellular matrix disassembly and organization, neuron projection development, protein complex assembly	1	-	-	1	[Blauw, Al-Chalabi et al. 2010]
LAMC2	Cell adhesion, extracellular matrix disassembly and organization	1	-	-	1	[Blauw, Al-Chalabi et al. 2010]
LIMS3	-	1	-	1	-	[Blauw, Barnes et al. 2008]
LIPC	Developmental growth, response to hypoxia, cholesterol transport, small molecule metabolic process	1	-	1	-	[Blauw, Al-Chalabi et al. 2010]
LIPF	Lipid metabolic process, oxidation-reduction process	1	-	-	1	[Blauw, Al-Chalabi et al. 2010]
LIPK	Lipid catabolic and metabolic process	1	-	-	1	[Blauw, Al-Chalabi et al. 2010]
LRRC16A	Actin filament network formation and organization, cell migration	1	-	-	1	[Blauw, Al-Chalabi et al. 2010]
LRRC30	-	1	-	-	1	[Wain, Pedroso et al. 2009]
LRRTM1	Cytokine-mediated signaling pathway, exploration behavior, locomotory behavior, long-term synaptic potentiation, regulation of JAK-STAT cascade, regulation of protein kinase activity, regulation of synapse assembly and organization	1	-	-	1	[Blauw, Al-Chalabi et al. 2010]
LYRM4	Small molecule metabolic process	1	-	1	-	[Blauw, Al-Chalabi et al. 2010]
LZIC	Response to ionizing radiation	1	-	1	-	[Blauw, Al-Chalabi et al. 2010]
LZTS3	Protein homooligomerization	1	-	1	-	[Blauw, Al-Chalabi et al. 2010]
MAD2L1	Regulation of apoptotic process, regulation of ubiquitin-protein ligase activity, regulation of cell cycle, signal transduction	1	-	1	-	[Blauw, Barnes et al. 2008]
MADD	Apoptotic process, regulation of cell cycle, signaling pathway	1	-	1	-	[Blauw, Al-Chalabi et al. 2010]
MANF	Response to unfolded protein	1	-	-	1	[Blauw, Al-Chalabi et al. 2010]
MAT1A	Metabolic process	1	-	-	1	[Blauw, Al-Chalabi et al. 2010]
MED11	Protein ubiquitination, regulation of transcription	1	-	1	-	[Blauw, Al-Chalabi et al. 2010]
MED13L	Regulation of transcription	1	-	1	-	[Blauw, Al-Chalabi et al. 2010]
MEPE	Skeletal system development	1	-	-	1	[Blauw, Al-Chalabi et al. 2010]
MFSD4B	Ion transport	1	-	-	1	[Pamphlett, Morahan et al. 2011]
MRO	-	1	-	-	1	[Blauw, Al-Chalabi et al. 2010]
MYO15A	Locomotory behavior, metabolic process, sensory perception of sound	1	-	1	-	[Blauw, Al-Chalabi et al. 2010]
N4BP2L1	-	1	-	1	-	[Blauw, Al-Chalabi et al. 2010]

N4BP2L2	-	1	-	1	-	[Blauw, Al-Chalabi et al. 2010]
N4BP3	-	1	-	1	-	[Blauw, Barnes et al. 2008]
NAPG	Protein complex assembly, protein stabilization, protein transport	1	-	-	1	[Blauw, Al-Chalabi et al. 2010]
NAT2	Aging, metabolic process	1	-	1	-	[Blauw, Barnes et al. 2008]
NCOA7	Regulation of transcription	1	-	1	-	[Blauw, Barnes et al. 2008]
NDNF	Angiogenesis, cell growth, cellular response to hypoxia, extracellular matrix organization, regulation of neuron apoptotic process, nervous system development, neuron migration, signal transduction	1	-	1	-	[Blauw, Barnes et al. 2008]
NDUFC1	Cellular metabolic process, mitochondrial electron transport, oxidation-reduction process	1	-	1	-	[Blauw, Al-Chalabi et al. 2010]
NEURL1	Notch signaling pathway, brain development, regulation of apoptotic process, regulation of epidermal growth factor-activated receptor activity, regulation of long-term neuronal synaptic plasticity, positive regulation of synapse maturation, protein ubiquitination, regulation of translation, skeletal muscle tissue development	1	-	-	1	[Blauw, Al-Chalabi et al. 2010]
NHP2	RNA processing	1	-	1	-	[Blauw, Barnes et al. 2008]
NME5	Cell differentiation, regulation of oxidative stress-induced intrinsic apoptotic signaling pathway, metabolic process	1	-	-	1	[Blauw, Al-Chalabi et al. 2010]
NMNAT1	NAD biosynthetic process	1	-	1	-	[Blauw, Al-Chalabi et al. 2010]
NPPC	Regulation of DNA metabolic process, regulation of cell proliferation, regulation of cell cycle, response to hypoxia	1	-	-	1	[Blauw, Al-Chalabi et al. 2010]
NSA2	RNA processing	1	-	1	-	[Blauw, Al-Chalabi et al. 2010]
NUDT16P1	-	1	-	1	-	[Blauw, Barnes et al. 2008]
NUPL2	Metabolic process, gene expression, post-translational protein modification, tRNA processing, transport	1	-	1	-	[Blauw, Barnes et al. 2008]
OR1A1	G-protein coupled receptor signaling pathway, positive regulation of cytokinesis, response to stimulus, sensory perception of smell, signal transduction	1	-	-	1	[Blauw, Al-Chalabi et al. 2010]
OR1A2	G-protein coupled receptor signaling pathway, positive regulation of cytokinesis, response to stimulus, sensory perception of smell, signal transduction	1	-	-	1	[Blauw, Al-Chalabi et al. 2010]
OR1D2	G-protein coupled receptor signaling pathway, positive regulation of cytokinesis, response to stimulus, sensory perception of smell, signal transduction, chemotaxis	1	-	-	1	[Blauw, Al-Chalabi et al. 2010]
OR1D4	G-protein coupled receptor signaling pathway, positive regulation of cytokinesis, response to stimulus, sensory perception of smell, signal transduction	1	-	-	1	[Blauw, Al-Chalabi et al. 2010]
OR1D5	G-protein coupled receptor signaling pathway, positive regulation of cytokinesis, response to stimulus, sensory perception of smell, signal transduction	1	-	-	1	[Blauw, Al-Chalabi et al. 2010]
OR1G1	G-protein coupled receptor signaling pathway, positive regulation of cytokinesis, response to stimulus, sensory perception of smell, signal transduction	1	-	-	1	[Blauw, Al-Chalabi et al. 2010]
OR51H1	-	1	-	1	-	[Blauw, Barnes et al. 2008]

OR51S1	G-protein coupled receptor signaling pathway, response to olfactory stimulus, signal transduction	1	-	1	-	[Blauw, Barnes et al. 2008]
OR51T1	G-protein coupled receptor signaling pathway, response to olfactory stimulus, signal transduction	1	-	1	-	[Blauw, Barnes et al. 2008]
OR52R1	G-protein coupled receptor signaling pathway, response to olfactory stimulus, signal transduction	1	-	1	-	[Blauw, Barnes et al. 2008]
OSBPL3	Lipid transport	1	-	-	1	[Blauw, Al-Chalabi et al. 2010]
OXT	Regulation of synapse assembly, regulation of synaptic transmission, response to mechanical stimulus, signal transduction, sleep, social behavior	1	-	1	-	[Blauw, Al-Chalabi et al. 2010]
P2RX7	NAD transport, T cell homeostasis and proliferation, apoptotic signaling pathway, ion transmembrane transport, cell morphogenesis, cell surface receptor signaling pathway, defense response, membrane depolarization, regulation of cytoskeleton organization, regulation of mitochondrial depolarization, protein processing, synaptic vesicle exocytosis	1	-	-	1	[Blauw, Al-Chalabi et al. 2010]
PALM2	Regulation of cell shape	1	-	1	-	[Blauw, Al-Chalabi et al. 2010]
PAMR1	Proteolysis	1	-	1	-	[Blauw, Al-Chalabi et al. 2010]
PAPPA	Metabolic process	1	-	-	1	[Blauw, Al-Chalabi et al. 2010]
PARP16	Signaling protein activity involved in unfolded protein response, regulation of cell death	1	-	1	-	[Blauw, Al-Chalabi et al. 2010]
PBXIP1	Cell differentiation, regulation of transcription	1	-	1	-	[Blauw, Al-Chalabi et al. 2010]
PCGF6	Regulation of transcription	1	-	-	1	[Blauw, Al-Chalabi et al. 2010]
PCSK1	Cell-cell signaling, cellular protein metabolic process, proteolysis	1	-	-	1	[Blauw, Al-Chalabi et al. 2010]
PDE5A	Blood coagulation, metabolic process, nervous system development, regulation of apoptotic process, response to hypoxia, short-term memory, signal transduction	1	-	1	-	[Blauw, Al-Chalabi et al. 2010]
PDGFRA	Axon guidance, cell chemotaxis, cell migration, signaling pathway, extracellular matrix organization, innate immune response, protein phosphorylation, regulation of actin cytoskeleton reorganization, response to cytokine, response to hormone, response to hyperoxia, regulation of gene expression, signaling pathway	1	1	-	-	[Pamphlett, Morahan et al. 2011]
PDLIM1	Regulation of transcription, response to oxidative stress	1	-	-	1	[Blauw, Al-Chalabi et al. 2010]
PELP1	Cellular response to estrogen stimulus, positive regulation of transcription	1	-	1	-	[Blauw, Al-Chalabi et al. 2010]
PITPNM3	Phosphatidylinositol metabolic process, phospholipid transport	1	-	-	1	[Blauw, Al-Chalabi et al. 2010]
PLA2R1	Cytokine production, endocytosis, response to oxidative stress, regulation of DNA damage response, signal transduction, receptor-mediated endocytosis	1	-	-	1	[Blauw, Al-Chalabi et al. 2010]
PLD4	Lipid metabolic process	1	-	-	1	[Blauw, Al-Chalabi et al. 2010]
PMVK	Lipid metabolic process, phosphorylation	1	-	1	-	[Blauw, Al-Chalabi et al. 2010]
POU1F1	B cell differentiation, regulation of cell proliferation, regulation of transcription	1	-	-	1	[Blauw, Al-Chalabi et al. 2010]
PPA2	Metabolic process, protein dephosphorylation	1	-	-	1	[Blauw, Al-Chalabi et al. 2010]
PPP1R16B	Regulation of protein dephosphorylation	1	-	1	-	[Blauw, Al-Chalabi et al. 2010]

PRDM5	Histone deacetylation, methylation, mitotic cell cycle, regulation of transcription	1	-	1	-	[Blauw, Barnes et al. 2008]
PRKD1	Golgi organization, Golgi vesicle transport, signal transduction, angiogenesis, apoptotic process, cell differentiation, cell proliferation, cellular response to oxidative stress, immune system process, inflammatory response, regulation of endocytosis, nervous system development, phosphorylation, chemotaxis, small molecule metabolic process	1	-	1	-	[Blauw, Al-Chalabi et al. 2010]
PRMT3	Dendritic spine morphogenesis, regulation of protein ubiquitination, regulation of transcription	1	-	-	1	[Blauw, Al-Chalabi et al. 2010]
PRUNE2	Apoptotic process, catabolic process	1	-	-	1	[Blauw, Al-Chalabi et al. 2010]
PTGER3	G-protein coupled receptor signaling pathway, cell death, regulation of calcium ion transmembrane transport, regulation of gene expression, signal transduction, transcription	1	-	-	1	[Blauw, Al-Chalabi et al. 2010]
PTPRM	Cell adhesion, dephosphorylation, neuron projection development, signal transduction	1	-	1	-	[Blauw, Barnes et al. 2008]
PTPRT	Cell adhesion, dephosphorylation, signal transduction	1	1	-	-	[Pamphlett, Morahan et al. 2011]
PXDN	Cellular oxidant detoxification, extracellular matrix organization, immune response	1	-	-	1	[Blauw, Al-Chalabi et al. 2010]
PYROXD1	Oxidation-reduction process	1	-	1	-	[Blauw, Al-Chalabi et al. 2010]
RAB33B	Protein transport, signal transduction	1	-	1	-	[Blauw, Al-Chalabi et al. 2010]
RAB9A	Signal transduction, protein transport	1	-	-	1	[Wain, Pedroso et al. 2009]
RARB	Gene expression, regulation of apoptotic process, regulation of cell proliferation, regulation of neuron differentiation, transcription	1	-	1	-	[Pamphlett, Morahan et al. 2011]
RARG	Wnt signaling pathway, gene expression, regulation of apoptotic process, regulation of cell differentiation, regulation of cell proliferation, regulation of gene expression	1	-	1	-	[Blauw, Barnes et al. 2008]
RBM15B	RNA splicing, mRNA processing, transcription	1	-	-	1	[Blauw, Al-Chalabi et al. 2010]
RBP7	Transport	1	-	1	-	[Blauw, Al-Chalabi et al. 2010]
RECQL	DNA repair, DNA replication	1	-	1	-	[Blauw, Al-Chalabi et al. 2010]
REM1	Signal transduction	1	-	-	1	[Blauw, Al-Chalabi et al. 2010]
RERGL	Signal transduction	1	-	-	1	[Blauw, Al-Chalabi et al. 2010]
RHAG	Cellular ion homeostasis, metabolic process, transmembrane transport	1	-	1	-	[Blauw, Al-Chalabi et al. 2010]
RITA1	Notch signaling pathway, regulation of transcription, nervous system development, neurogenesis	1	-	1	-	[Blauw, Al-Chalabi et al. 2010]
RMND5B	-	1	-	1	-	[Blauw, Barnes et al. 2008]
RNASE10	Regulation of cell-cell adhesion	1	-	-	1	[Blauw, Al-Chalabi et al. 2010]
RNGTT	RNA processing, transcription, metabolic process	1	-	-	1	[Blauw, Al-Chalabi et al. 2010]
RPL9	Cellular protein metabolic process, cellular response to nerve growth factor stimulus, gene expression	1	-	-	1	[Blauw, Al-Chalabi et al. 2010]
RPS10	Cellular protein metabolic process, cellular response to nerve growth factor stimulus, cytoplasmic translation, gene expression	1	-	-	1	[Blauw, Al-Chalabi et al. 2010]
RPS7	Cell differentiation, cellular protein metabolic process, gene expression	1	-	-	1	[Blauw, Al-Chalabi et al. 2010]

RSG1	Cell projection organization, cellular protein localization, exocytosis, protein transport, signal transduction	1	-	-	1	[Blauw, Al-Chalabi et al. 2010]
RSPH4A	Axoneme assembly, cilium movement	1	-	1	-	[Blauw, Al-Chalabi et al. 2010]
RWDD1	-	1	-	1	-	[Blauw, Al-Chalabi et al. 2010]
RXFP2	Regulation of apoptotic process, signal transduction	1	-	1	-	[Blauw, Barnes et al. 2008]
SAR1AP3	-	1	-	1	-	[Blauw, Barnes et al. 2008]
**SDK1**	Cell adhesion, synapse assembly	1	-	1	-	[Pamphlett, Morahan et al. 2011]
SERPINB3	Autocrine signaling, regulation of cell migration, regulation of cell proliferation	1	-	1	-	[Blauw, Al-Chalabi et al. 2010]
SESTD1	-	1	-	1	-	[Pamphlett, Morahan et al. 2011]
SGTA	Regulation of ER-associated ubiquitin-dependent protein catabolic process	1	-	-	1	[Blauw, Al-Chalabi et al. 2010]
SLC12A8	Ion transport	1	-	1	-	[Blauw, Barnes et al. 2008]
**SLC1A7**	L-glutamate transmembrane transport, neurotransmitter secretion, synaptic transmission	1	-	-	1	[Schoichet, Waibel et al. 2009]
SLC24A1	Ion transport, cellular calcium ion homeostasis	1	-	1	-	[Blauw, Al-Chalabi et al. 2010]
SLC25A30	positive regulation of ER-associated ubiquitin-dependent protein catabolic process	1	-	-	1	[Blauw, Al-Chalabi et al. 2010]
SLC26A6	Angiotensin-activated signaling pathway, transmembrane transport	1	-	1	-	[Blauw, Barnes et al. 2008]
SLC34A2	Aging, cellular protein metabolic process, ion transport	1	-	-	1	[Blauw, Al-Chalabi et al. 2010]
SLC5A9	Ion transport	1	-	1	-	[Blauw, Al-Chalabi et al. 2010]
SLCO4C1	Cell differentiation, ion transport	1	-	1	-	[Blauw, Barnes et al. 2008]
SLK	Apoptotic process, cytoplasmic microtubule organization, phosphorylation, regulation of cell migration, regulation of focal adhesion assembly, regulation of mitotic cell cycle, signal transduction	1	-	1	-	[Blauw, Al-Chalabi et al. 2010]
SNX19	Exocytosis, protein transport, transport	1	-	-	1	[Blauw, Al-Chalabi et al. 2010]
SNX29	Cell communication	1	1	-	-	[Pamphlett, Morahan et al. 2011]
SORCS3	Learning, memory, neuropeptide signaling pathway, regulation of long term synaptic depression	1	-	-	1	[Blauw, Al-Chalabi et al. 2010]
SPATA1	-	1	-	-	1	[Blauw, Al-Chalabi et al. 2010]
SPNS2	Lipid transport, regulation of immune response, transmembrane transport, transport	1	-	-	1	[Blauw, Al-Chalabi et al. 2010]
SPOCK1	Cell adhesion, central nervous system neuron differentiation, nervous system development, neurogenesis, neuron migration, regulation of cell growth, signal transduction	1	-	-	1	[Pamphlett, Morahan et al. 2011]
SRP68	Cellular protein metabolic process, gene expression, translation	1	-	1	-	[Blauw, Barnes et al. 2008]
SSX2IP	Actin cytoskeleton organization, cell adhesion, regulation of cell motility	1	-	-	1	[Blauw, Al-Chalabi et al. 2010]
ST3GAL6	Cellular response to interleukin-6, metabolic process	1	1	-	-	[Blauw, Al-Chalabi et al. 2010]
ST6GALNAC4	Cellular protein metabolic process, glycolipid metabolic process, post-translational protein modification	1	-	-	1	[Blauw, Al-Chalabi et al. 2010]
ST8SIA6	Cellular protein metabolic process	1	-	1	-	[Blauw, Al-Chalabi et al. 2010]
STARD13	Angiogenesis, cell cycle, signal transduction	1	-	1	-	[Blauw, Al-Chalabi et al. 2010]

STK39	Intracellular signal transduction, ion transmembrane transporter activity, regulation of protein phosphorylation, regulation of apoptotic process, regulation of inflammatory response, regulation of ion homeostasis, regulation of mitotic cell cycle, response to stress	1	-	1	-	[Blauw, Al-Chalabi et al. 2010]
SYN3	Metabolic process, neurotransmitter secretion, regulation of synaptic transmission	1	-	1	-	[Blauw, Al-Chalabi et al. 2010]
SYT8	Exocytosis	1	-	1	-	[Blauw, Barnes et al. 2008]
SZRD1	-	1	-	-	1	[Blauw, Al-Chalabi et al. 2010]
TBC1D3B	Regulation of GTPase activity	1	-	-	1	[Pamphlett, Morahan et al. 2011]
TCERG1	Regulation of transcription	1	-	1	-	[Blauw, Al-Chalabi et al. 2010]
TGFBR3	Cell growth, cell migration, immune response, regulation of apoptotic process, regulation of cellular regulation of gene expression, regulation of cell proliferation, protein complex assembly, regulation of protein binding, response to hypoxia,signal transduction	1	-	1	-	[Pamphlett, Morahan et al. 2011]
THADA	Death receptor signaling	1	-	1	-	[Blauw, Barnes et al. 2008]
THOC1	RNA processing, transcription, signal transduction	1	-	-	1	[Blauw, Al-Chalabi et al. 2010]
THOP1	Intracellular signal transduction, proteolysis	1	-	-	1	[Blauw, Al-Chalabi et al. 2010]
TICAM2	Apoptotic signaling pathway, immune system process, inflammatory response, signal transduction	1	-	1	-	[Blauw, Al-Chalabi et al. 2010]
TIMP2	Aging, nervous system development, extracellular matrix disassembly and organization, signal transduction, regulation of cell proliferation, regulation of mitotic cell cycle, regulation of peptidase activity, regulation of neuron differentiation	1	-	1	-	[Blauw, Al-Chalabi et al. 2010]
TLN2	Cell adhesion, cell-cell junction assembly, cytoskeletal anchoring at plasma membrane	1	-	-	1	[Blauw, Al-Chalabi et al. 2010]
TM4SF5	Signal transduction, cell development, cell growth, cell motility	1	-	1	-	[Blauw, Al-Chalabi et al. 2010]
TMED7	Cellular protein metabolic process, membrane organization, post-translational protein modification, protein transport	1	-	1	-	[Blauw, Al-Chalabi et al. 2010]
TMEM155	-	1	-	1	-	[Blauw, Barnes et al. 2008]
TMEM156	-	1	-	1	-	[Blauw, Al-Chalabi et al. 2010]
TMEM184C	Transport	1	-	-	1	[Blauw, Al-Chalabi et al. 2010]
TMEM89	-	1	-	1	-	[Blauw, Barnes et al. 2008]
TNIP3	Inflammatory response	1	-	1	-	[Blauw, Barnes et al. 2008]
TNKS2	Wnt signaling pathway, protein polyubiquitination	1	1	-	-	[Pamphlett, Morahan et al. 2011]
TNNI2	Regulation of transcription, regulation of muscle contraction	1	-	1	-	[Blauw, Barnes et al. 2008]
TNS1	Fibroblast migration, intracellular signal transduction	1	-	1	-	[Blauw, Al-Chalabi et al. 2010]
TOPBP1	DNA metabolic process, DNA repair, cellular response to DNA damage stimulus	1	-	-	1	[Blauw, Al-Chalabi et al. 2010]
TPCN1	Ion transmembrane transport, membrane depolarization during action potential, positive regulation of autophagy	1	-	1	-	[Blauw, Al-Chalabi et al. 2010]
TPT1	Calcium ion transport, cell proliferation, cellular calcium ion homeostasis, regulation of apoptotic process, regulation of ectoderm development	1	-	-	1	[Blauw, Al-Chalabi et al. 2010]

**TRDN**	Cellular calcium ion homeostasis, cytoplasmic microtubule organization, endoplasmic reticulum membrane organization, muscle contraction, transmembrane transport	1	-	-	1	[Schoichet, Waibel et al. 2009]
TRIM45	Bone development	1	-	1	-	[Blauw, Al-Chalabi et al. 2010]
TRIM71	Cell cycle, fibroblast growth factor receptor signaling pathway, gene silencing by RNA, miRNA metabolic process, neural tube development, protein ubiquitination, regulation of neural precursor cell proliferation, regulation of protein metabolic process	1	-	-	1	[Blauw, Al-Chalabi et al. 2010]
TRMT11	Gene expression, methylation, RNA processing	1	-	1	-	[Blauw, Al-Chalabi et al. 2010]
TRPC3	Axon guidance, ion transmembrane transport	1	-	1	-	[Blauw, Barnes et al. 2008]
TSEN15	Gene expression, RNA processing	1	-	-	1	[Blauw, Al-Chalabi et al. 2010]
TSPAN14	Cell surface receptor signaling pathway, positive regulation of Notch signaling pathway, protein maturation	1	-	-	1	[Blauw, Al-Chalabi et al. 2010]
TTC23	Response to endoplasmic reticulum stress	1	-	-	1	[Blauw, Al-Chalabi et al. 2010]
TTF2	Transcription, RNA splicing, mRNA processing	1	-	1	-	[Blauw, Al-Chalabi et al. 2010]
TXNRD3NB	-	1	-	-	1	[Blauw, Al-Chalabi et al. 2010]
UBE4B	Apoptotic process, cellular protein catabolic process, neuron projection development, protein polyubiquitination	1	-	1	-	[Blauw, Al-Chalabi et al. 2010]
UFSP2	Proteolysis, regulation of intracellular estrogen receptor signaling pathway	1	-	-	1	[Blauw, Al-Chalabi et al. 2010]
UQCRC1	Cellular metabolic process, ion transmembrane transport, mitochondrial electron transport, oxidation-reduction process, protein processing	1	-	1	-	[Blauw, Barnes et al. 2008]
USP14	Regulation of ER-associated ubiquitin-dependent protein catabolic process, regulation of endopeptidase activity, proteolysis, regulation of chemotaxis, synaptic transmission, ubiquitin-dependent protein catabolic process	1	-	-	1	[Blauw, Al-Chalabi et al. 2010]
USP29	Proteolysis, ubiquitin-dependent protein catabolic process	1	-	-	1	[Blauw, Al-Chalabi et al. 2010]
USP53	Protein deubiquitination, sensory perception of sound	1	-	1	-	[Blauw, Al-Chalabi et al. 2010]
VIM	Signal transduction, aging, apoptotic process, astrocyte development, intermediate filament organization, regulation of neuron projection development, regulation of gene expression, glial cell proliferation, regulation of Schwann cell migration, regulation of axonogenesis	1	-	1	-	[Blauw, Al-Chalabi et al. 2010]
VPS33B	Autophagosome maturation, endosome organization, transport, exocytosis, vesicle-mediated transport	1	-	-	1	[Blauw, Al-Chalabi et al. 2010]
VTCN1	Immune response	1	-	1	-	[Blauw, Al-Chalabi et al. 2010]
VWA9	-	1	-	1	-	[Blauw, Al-Chalabi et al. 2010]
WBP11	RNA processing, RNA splicing	1	-	1	-	[Blauw, Al-Chalabi et al. 2010]
WNT8A	Wnt signaling pathway, cell morphogenesis, endoderm development, neuron differentiation, regulation of protein localization, regulation of transcription	1	-	-	1	[Blauw, Al-Chalabi et al. 2010]
XCL1	Cell-cell signaling, chemotaxis, immune response, inflammatory response, regulation of transcription, signal transduction	1	-	1	-	[Blauw, Al-Chalabi et al. 2010]
XCL2	Cell-cell signaling, chemotaxis, immune response, inflammatory response, regulation of transcription, signal transduction	1	-	1	-	[Blauw, Al-Chalabi et al. 2010]

YPEL2	-	1	-	-	1	[Blauw, Al-Chalabi et al. 2010]
ZCCHC2	-	1	-	1	-	[Blauw, Al-Chalabi et al. 2010]
ZDHHC24	Metabolic process	1	-	1	-	[Blauw, Barnes et al. 2008]
ZER1	Protein ubiquitination	1	-	1	-	[Wain, Pedroso et al. 2009]
ZMYND15	Cell differentiation, regulation of transcription	1	-	1	-	[Blauw, Al-Chalabi et al. 2010]
ZNF137P	Regulation of transcription	1	-	1	-	[Blauw, Barnes et al. 2008]
ZNF554	-	1	-	-	1	[Blauw, Al-Chalabi et al. 2010]
ZNF555	-	1	-	-	1	[Blauw, Al-Chalabi et al. 2010]
ZNF556	Transcription	1	-	-	1	[Blauw, Al-Chalabi et al. 2010]
ZNF600	Regulation of transcription	1	-	1	-	[Blauw, Barnes et al. 2008]
ZNF611	Regulation of transcription	1	-	1	-	[Blauw, Barnes et al. 2008]
ZNF642	Regulation of transcription	1	-	1	-	[Blauw, Barnes et al. 2008]
ZNF682	Transcription	1	-	-	1	[Blauw, Al-Chalabi et al. 2010]
ZNF684	Regulation of transcription	1	-	1	-	[Blauw, Barnes et al. 2008]
ZNF701	Regulation of transcription	1	-	1	-	[Blauw, Barnes et al. 2008]
ZNF740	Regulation of transcription	1	-	1	-	[Blauw, Barnes et al. 2008]
ZNF93	Transcription	1	-	-	1	[Blauw, Al-Chalabi et al. 2010]
ZUFSP	-	1	-	1	-	[Blauw, Al-Chalabi et al. 2010]
